# Vinylene-Bridged Cyclic Dipyrrin and BODIPY Trimers

**DOI:** 10.3390/ijms21218041

**Published:** 2020-10-28

**Authors:** Songlin Xue, Daiki Kuzuhara, Naoki Aratani, Hiroko Yamada

**Affiliations:** 1 School of Chemistry and Chemical Engineering, Jiangsu University, 301 Xuefu Road, Zhenjiang 212013, China; ricardoxue811@outlook.com; 2 Faculty of Science and Engineering, Iwate University, 4-3-5 Ueda, Morioka, Iwate 020-8551, Japan; 3 Division of Materials Science, Nara Institute of Science and Technology, 8916-5 Takayama-cho, Ikoma, Nara 630-0192, Japan; aratani@ms.naist.jp

**Keywords:** expanded porphyrin, boron complex, molecular configuration

## Abstract

Vinylene-bridged cyclic boron–difluoride complex of dipyrrin (BODIPY) trimers were successfully prepared from expanded dimethyl-vinylene bridged hexaphyrin(2.1.2.1.2.1) Me-Hex that has the structure of alternate dipyrrins and vinylene bridges. The hexaphyrin(2.1.2.1.2.1) Me-Hex can coordinate with boron ions to afford five kinds of cyclic BODIPYs given by step-by-step boron complexations. Crystal structures of all cyclic BODIPYs except for 3BF_2_-Me-Hex(b) formed non-planar structures. The theoretical calculation predicted that mono-/bis-boron cyclic BODIPYs show the intramolecular charge transfer (ICT) characteristics, whereas tri-boron cyclic BODIPYs have no ICT characteristics. Reflecting these electronic properties, tri-boron cyclic BODIPYs exhibit weak fluorescence in the red region, but mono-/bis-boron cyclic BODIPYs exhibit no emission. Vinylene bridged cyclic dipyrrin trimer Me-Hex is the novel porphyrinoid ligand allowed to control the boron coordination under different reaction conditions to form various boron complexes.

## 1. Introduction

**Figure 1:**
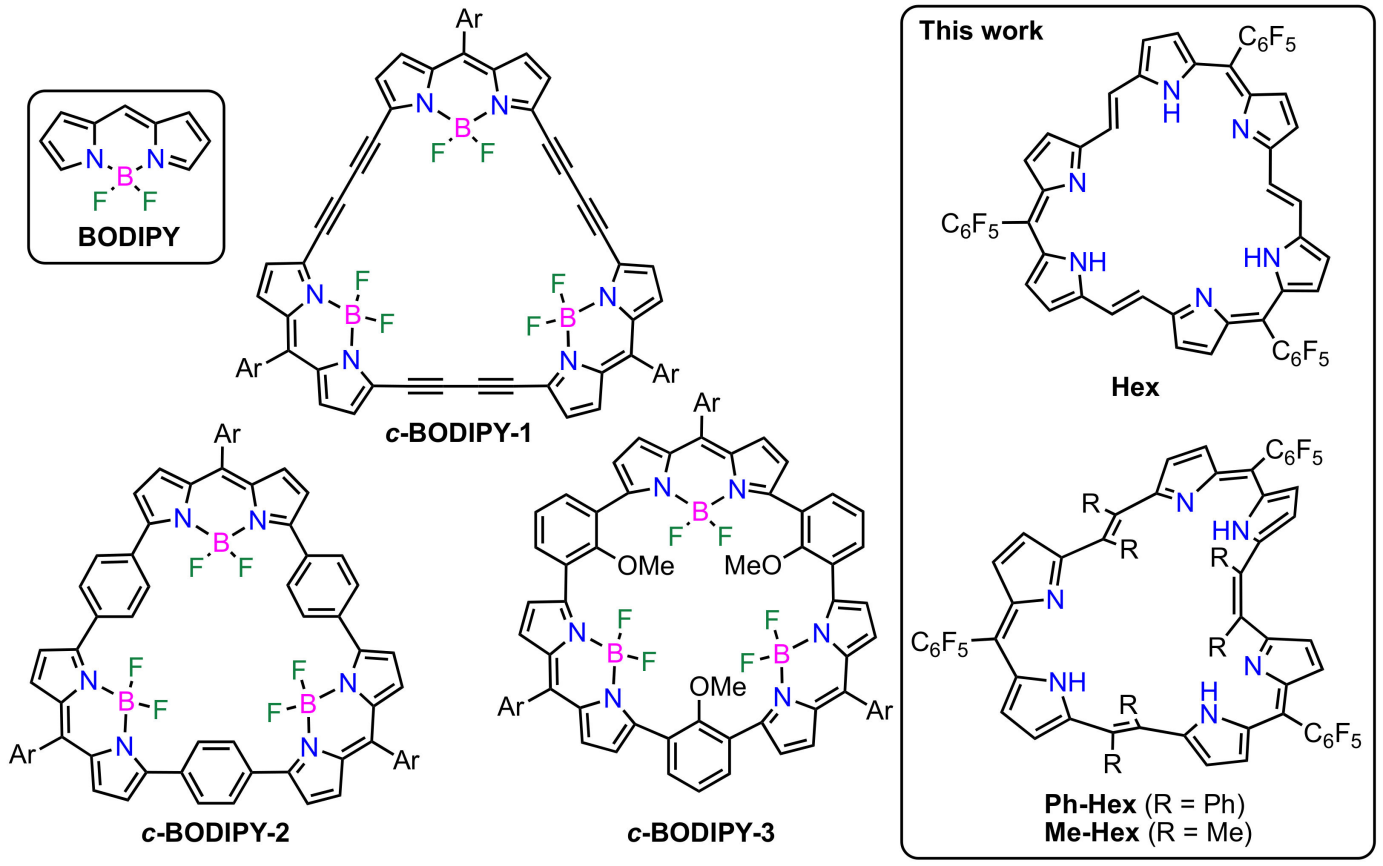
Chemical structures of boron–difluoride complex of dipyrrin (BODIPY) and cyclic BODIPY trimers with various linkages, and expanded porphyrins Hex, Ph-Hex, and Me-Hex.

A boron–difluoride complex of dipyrrin (BODIPY) is an outstanding fluorescent dye with high fluorescence quantum yield and high chemical robustness [1,2,3,4]. BODIPYs have been used for many applications such as optical materials, solar cells, biological imaging, and sensitizers for photodynamic therapy [5,6,7]. Among BODIPY derivatives, cyclic BODIPY oligomers show distinct electronic and optical properties such as unique luminescent and lasing properties, cation recognition, and switchable near infrared (NIR) responses [8,9,10,11,12,13,14,15]. Shinokubo and co-workers developed the synthesis of planar BODIPY dimer and trimer (*c*-BODIPY-1), linked through butadiyne by sila-Glaser coupling reaction (Figure 1) [9]. These cyclic BODIPYs are stable anti-aromatic compounds with NIR absorbing properties. Nabeshima and co-workers also reported a series of cyclic BODIPY oligomers linked through meta- and para-phenylenes (*c*-BODIPY-2 and *c*-BODIPY-3) (Figure 1) [11,12,13,14,15]. Their results show that the expanded porphyrins containing π-conjugated bridging units could be excellent precursors for the preparation of cyclic BODIPY oligomers. However, research on cyclic BODIPY oligomers is still limited due to few efficient synthesis methods [8,9,10,11,12,13,14,15]. Moreover, the relationship between the number of coordinated boron atoms and molecular configuration/optical properties of expanded porphyrins is still unclear.

Recently, we developed the synthesis of expanded porphyrins that include alternate vinylene bonds and dipyrrin units [16,17,18,19]. These expanded porphyrins are flexible and switchable molecular frameworks because of containing the vinylene bridges that can isomerize by external stimuli. In addition, molecular configurations and electronic properties can be tuned by substitution on vinylene bridges. The pristine *trans*-vinylene-bridged hexaphyrin(2.1.2.1.2.1), Hex, forms a planar structure with the aromatic property, whereas diphenyl-vinylene bridged hexaphyrin(2.1.2.1.2.1), Ph-Hex, forms the highly distorted structure with the non-aromatic property. Ph-Hex has bulky phenyl groups and mixed configurations on vinylene bridges, resulting in the formation of the distorted structure. Interestingly, the configuration of Ph-Hex is changed to the *cis*-vinylene-bridged figure-of-eight structure with the aromatic property by insertion of copper ions [18]. These results indicate that the expanded porphyrins having vinylene-bridges are curious ligands to make metal complexes accompanied by changes in molecular configurations and electronic properties. In addition, they include the dipyrrin unit that is a useful bidentate mono-anionic ligand capable of preparing various metal complexes, including BODIPYs.

In this paper, we report the synthesis, crystal structure, and optical properties of vinylene-bridged expanded porphyrins and boron complexes, using the dimethyl-vinylene bridged hexaphyrin(2.1.2.1.2.1), Me-Hex. We found that Me-Hex is a suitable ligand for the preparation of five boron complexes. Distorted and flexible structures lead to adjusting the coordination structure to boron ions, and the small methyl group allows sufficient space. We will discuss the reactivity of Me-Hex for inserting boron ions, crystal structures, and optical and electrochemical properties.

## 2. Results and Discussion

**Scheme 1:**
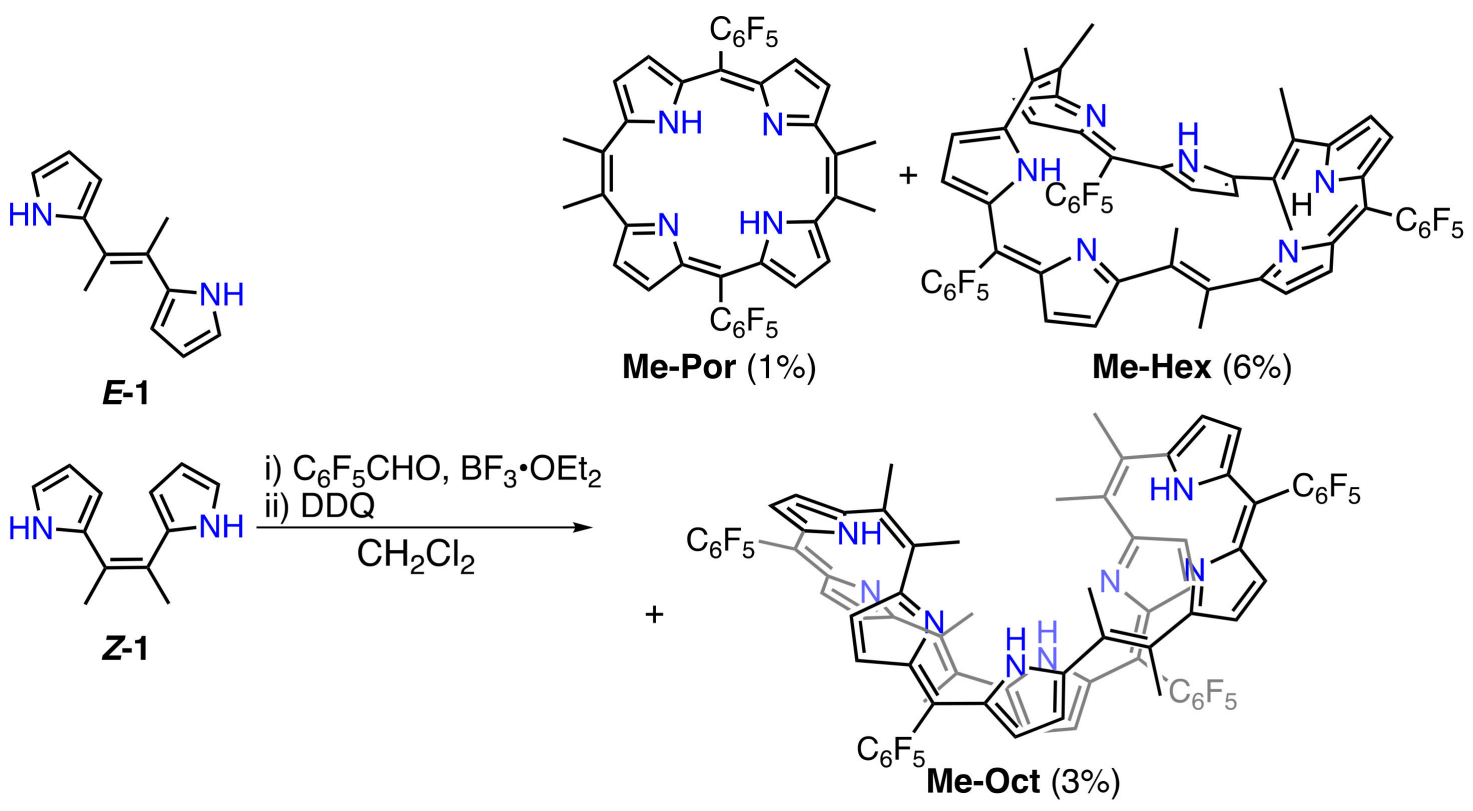
Synthesis of expanded porphyrins Me-Por, Me-Hex, and Me-Oct from E/Z-1.

Initially, we designed new hexaphyrin(2.1.2.1.2.1) with methyl groups on vinylene bridges as small substituents to make coordination spaces for preparing multi-boron complexes. The intermediate of dimethyl-dipyrrolylethenes (*E*/*Z*-1) was obtained from 2-acylpyrrole by McMurry coupling [11,12,13,14,15,16,17,18,20]. We and others reported that *E*/*Z*-dipyrroethenes undergo *cis*/*trans* isomerization in acidic conditions to prepare porphyrinoids [16,17,18,19,21]. Therefore, we used it as a mixture toward the next step. Treatment of *E*/*Z*-1 and pentafluorobenzaldehyde in the presence of BF_3_•OEt_2_ under condensation reactions and oxidation with 2,3-dichloro-5,6-dicyano-*p*-benzoquinone (DDQ) afforded three expanded porphyrins: Porphyrin(2.1.2.1), Me-Por, in 1%, hexaphyrin(2.1.2.1.2.1), Me-Hex, in 6%, and octaphyrin(2.1.2.1.2.1.2.1), Me-Oct, in 3% (Scheme 1). Characterization was conducted by matrix assisted Laser desorption/ionization-mass spectrometry (HR-MALDI-MS), X-ray crystallography, and ^1^H and ^19^F nuclear magnetic resonance (NMR) spectroscopy. In the HR-MALDI-MS, corresponding molecular ion peaks of Me-Por at *m*/*z* = 724.1683 (calculated for C_38_H_22_F_10_N_4_ = 724.1685 [M]^+^), Me-Hex at *m/z* = 1086.2522 (calculated for C_57_H_33_F_15_N_6_ = 1086.2527 [M]^+^), and Me-Oct at *m/z* = 1448.3364 (calculated for C_76_H_44_F_20_N_8_ = 1448.3370 [M]^+^) were observed, respectively (Figures S1–S3).

**Figure 2:**
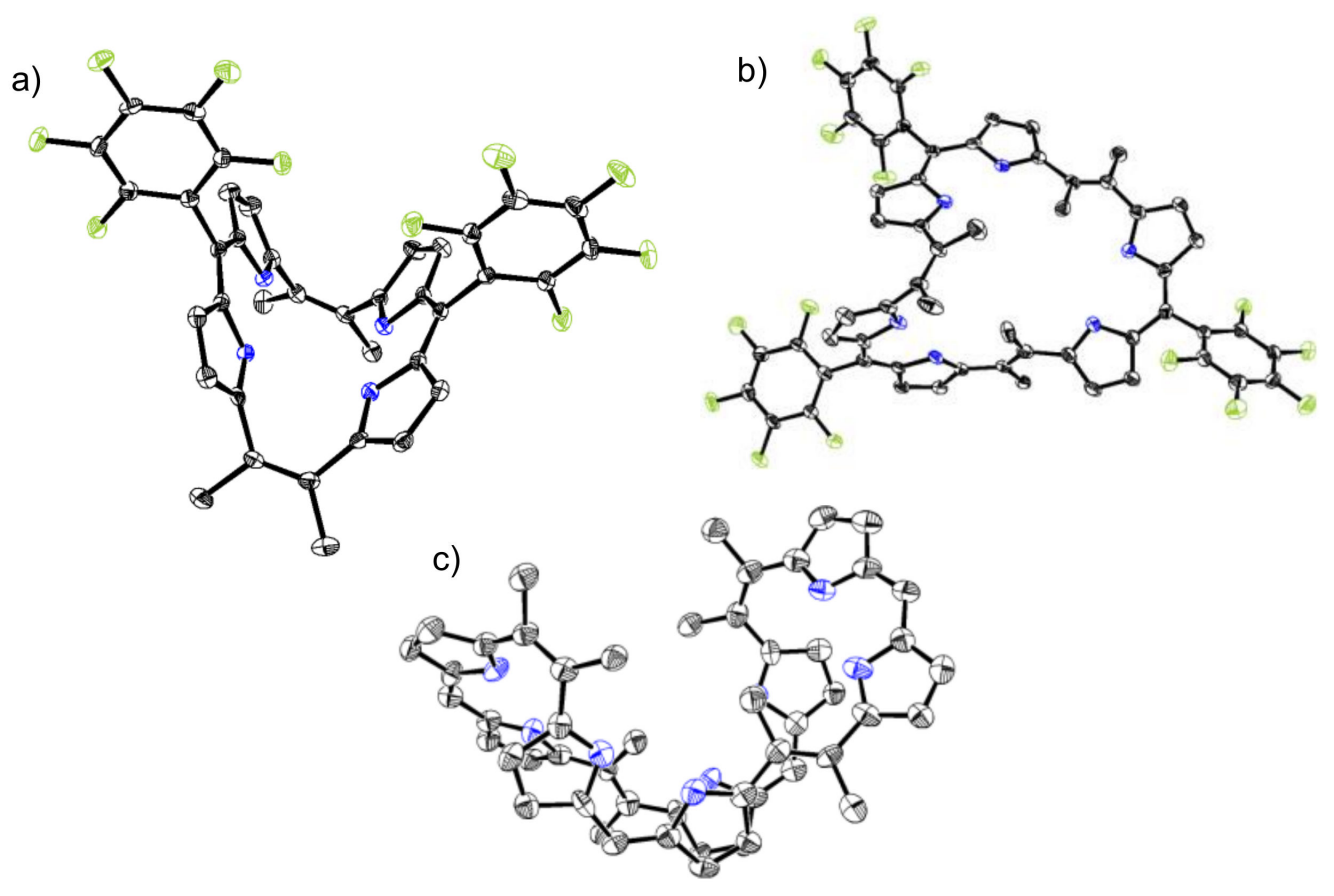
Crystal structures of (**a**) Me-Por, (**b**) Me-Hex, and (**c**) Me-Oct. Hydrogen atoms of three compounds and pentafluorophenyl groups of (**c**) were omitted for clarity. Thermal ellipsoids represent 50% probability.

The structures of these expanded porphyrins were determined by X-ray crystal structural analysis (Figure 2). Me-Por consists of two dipyrrin units connected through *cis*/*cis*-vinylene bridges, which make a saddle-shaped bent structure. This structure is similar to the structures of dibenzoporphyrin(2.1.2.1) and tetraphenylporphyrin(2.1.2.1) [16,18]. The compounds Me-Hex and Me-Oct also form distorted structures, mixing with *cis*- and *trans*-conformations at vinylene bridges. The compound Me-Hex comprises *cis*/*trans*/*trans* conformation at the vinylene bridges. The compound Me-Oct has a saddle-shaped distorted molecular structure consisting of *cis*/*trans*/*cis*/*trans* conformation at vinylene-bridges. All obtained porphyrins are categorized as having the same non-aromatic characteristic as Ph-Hex because of distorted structures [18].

**Figure 3:**
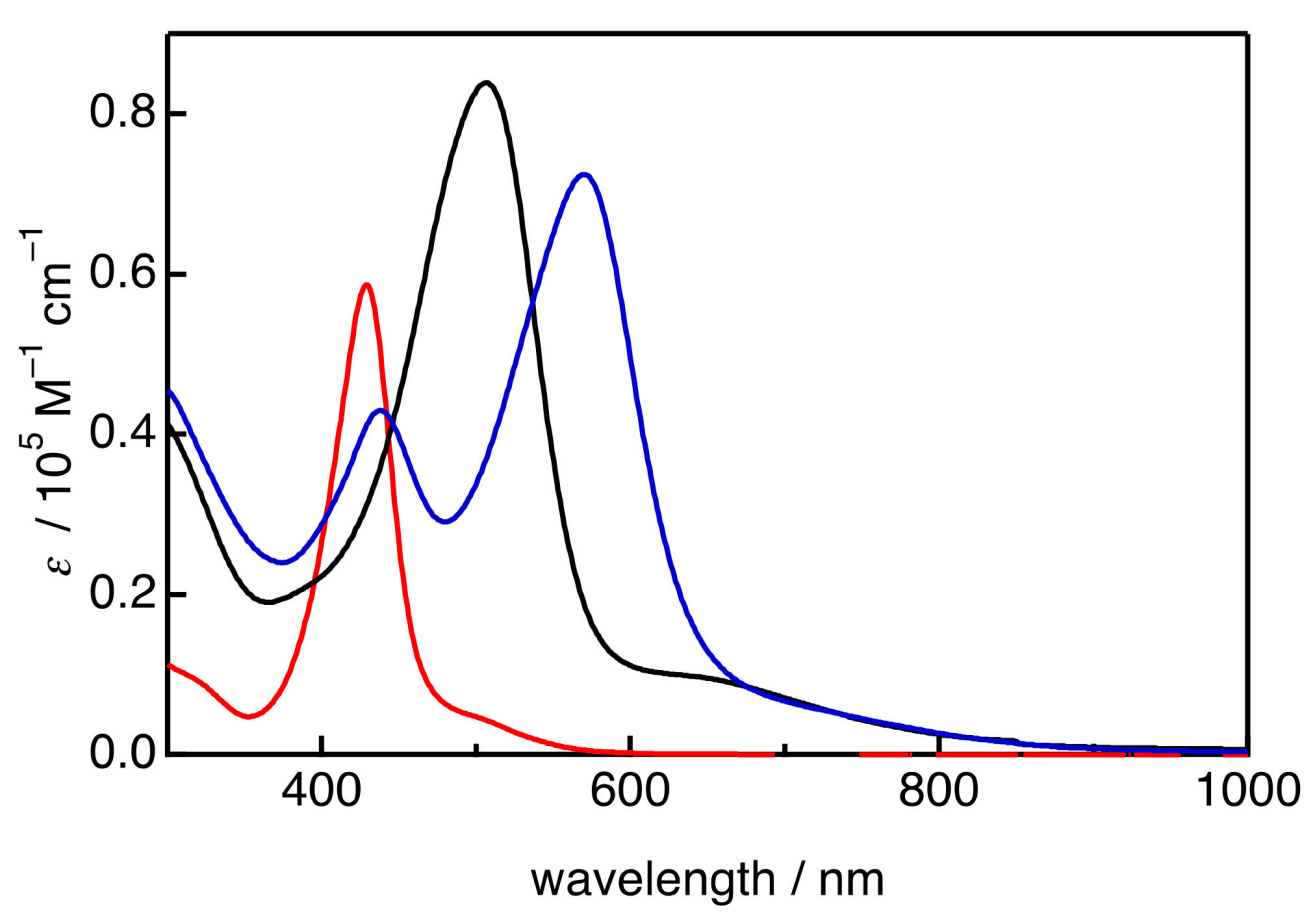
UV-vis absorption spectra of Me-Por (red), Me-Hex (black), and Me-Oct (blue) in CH_2_Cl_2_.

The UV-vis absorption spectra of Me-Por, Me-Hex, and Me-Oct were measured in CH_2_Cl_2_. These compounds show two distinct absorptions with strong and weak broad bands, reflecting their non-aromaticity (Figure 3) [16,17,18,19]. Maximum absorption peaks are observed at 432 nm for Me-Por, 500 nm for Me-Hex, and 574 nm for Me-Oct, which are gradually red-shifted by increasing in the ring size from Me-Por to Me-Oct as similar to expanded porphyrins. Their highest occupied molecular orbital (HOMO)-lowest unoccupied molecular orbital (LUMO) energy differences were estimated by density functional theory (DFT) calculations at B3LYP/6-31G* level (Figures S4 and S5). With increasing the ring size, the HOMO and LUMO energies increase and decrease, respectively. The HOMO levels of Me-Por, Me-Hex, and Me-Oct are −5.27 eV, −4.87 eV, and −4.78 eV, and the LUMO levels are −2.51 eV, −3.00 eV, and −3.04 eV, resulting in the decreasing the HOMO-LUMO difference.

**Scheme 2:**
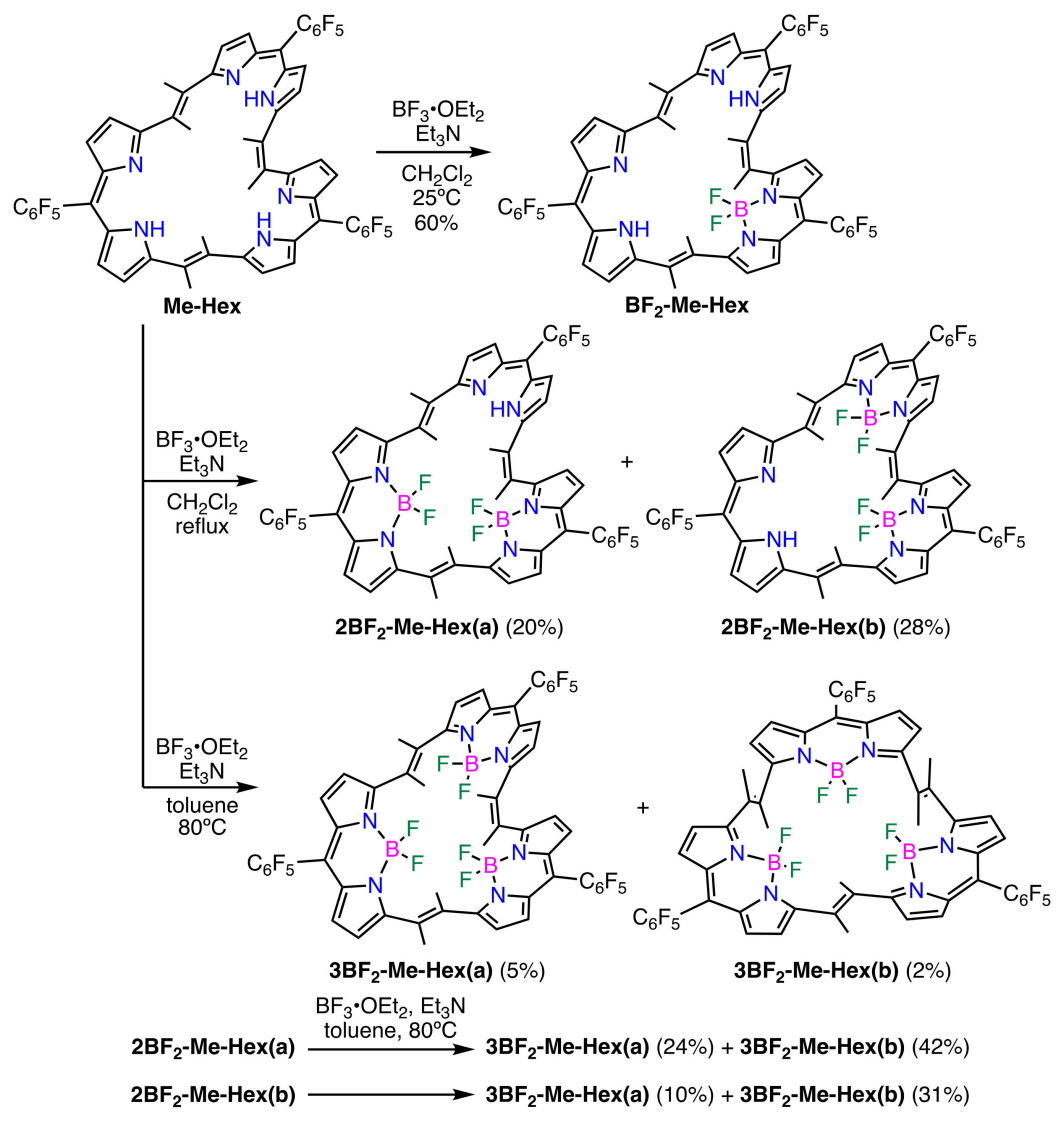
Synthesis of boron complexes of Me-Hex.

**Figure 4:**
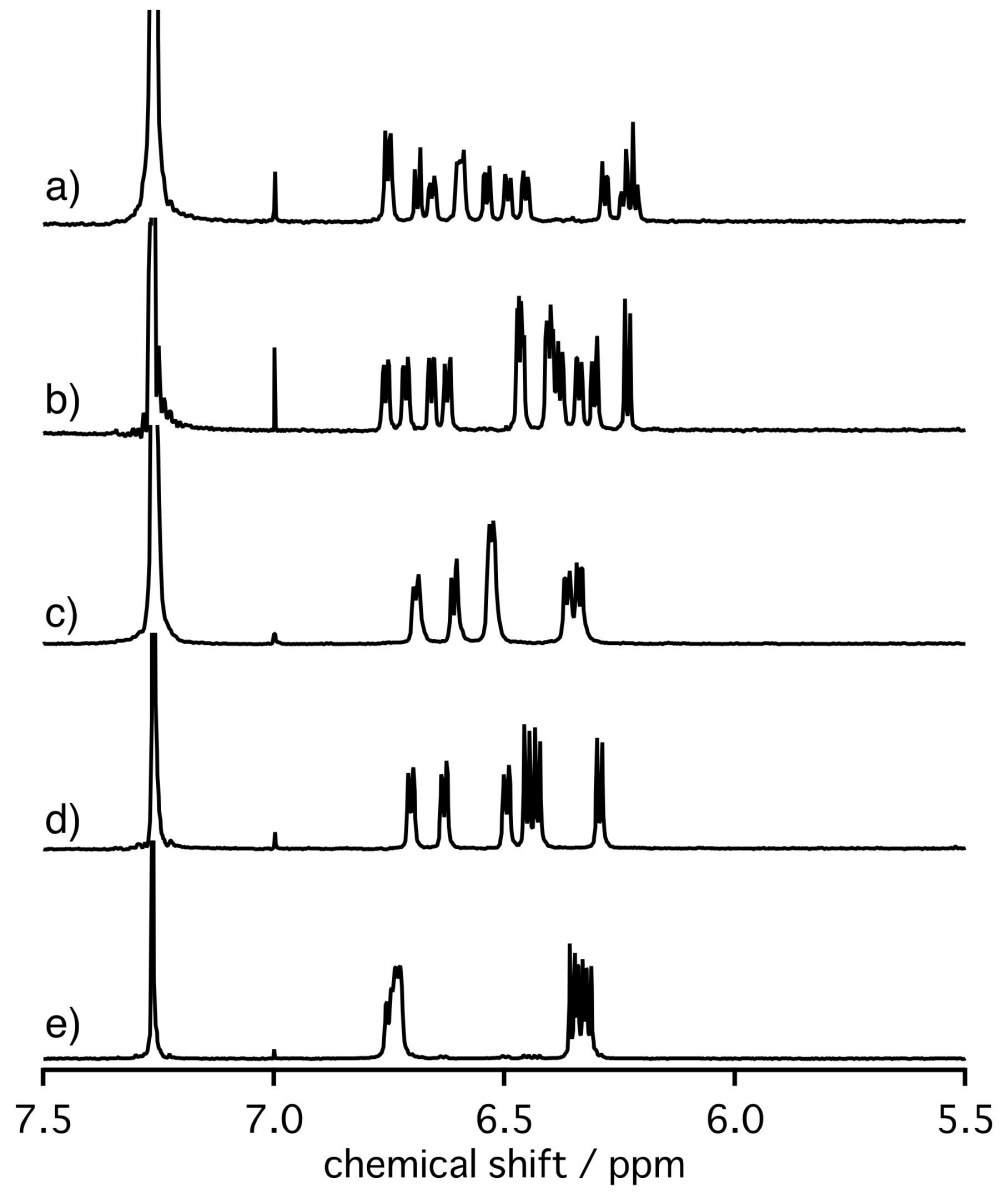
^1^H NMR spectra of (**a**) BF_2_-Me-Hex, (**b**) 2BF_2_-Me-Hex(a), (**c**) 2BF_2_-Me-Hex(b), (**d**) 3BF_2_-Me-Hex(a), and (**e**) 3BF_2_-Me-Hex(b) in CDCl_3_.

The differences in the formation of boron complexes of compound Me-Hex depending on the reaction conditions are summarized in Scheme 2. We optimized the reaction conditions of the boron complexation of Me-Hex. The treatment of Me-Hex with 40 eq. triethylamine (TEA) and 80 eq. BF_3_•OEt_2_ in CH_2_Cl_2_ at room temperature for 12 h mainly gave a mono-boron complex BF_2_-Me-Hex in 60% yield. ^1^H NMR spectrum of BF_2_-Me-Hex shows twelve sets of doublet peaks corresponding to pyrrolic *β*-protons and two broadened peaks at 13.26 and 13.07 ppm, corresponding to NH protons (Figure 4a). This result indicates that the first boron ion is coordinated into dipyrrin at the next to the *cis*-vinylene bridge as the asymmetric position. When an increase of reaction temperatures from room temperature to the reflux condition in CH_2_Cl_2_, two kinds of bis-boron complexes 2BF_2_-Me-Hex(a) and 2BF_2_-Me-Hex(b) that can be separated by silica gel column chromatography were generated in 20% and 28%, respectively (Scheme 2). The insertion positions of the second boron ion of 2BF_2_-Me-Hex(a) and 2BF_2_-Me-Hex(b) were confirmed by ^1^H NMR spectroscopy (Figure 4b,c) and X-ray crystallography (vide infra). 2BF_2_-Me-Hex(a) shows twelve sets of doublet peaks of pyrrolic *β*-protons and six methyl protons (Figure 4b). This structure is close to freebase Me-Hex and BF_2_-Me-Hex. In contrast, 2BF_2_-Me-Hex(b) shows the half number peak sets compared with 2BF_2_-Me-Hex(a) (Figure 4c). This means that 2BF_2_-Me-Hex(b) forms a 2-hold symmetric structure resulting in the formation of the bis-boron complex. Tri-boron complex was obtained from Me-Hex in the reaction with 40 eq. TEA and 120 eq. BF_3_•OEt_2_ in toluene at 80 °C. Unexpectedly, two types of tri-boron complexes, 3BF_2_-Me-Hex(a) and 3BF_2_-Me-Hex(b), were isolated in 5% and 2% yields, respectively, after separation by silica gel column chromatography. Additionally, these tri-boron complexes 3BF_2_-Me-Hex(a) and 3BF_2_-Me-Hex(b) were obtained from 2BF_2_-Me-Hex(a) and 2BF_2_-Me-Hex(b) under the same reaction conditions. The spectrum pattern of ^1^H NMR of 3BF_2_-Me-Hex(a) is similar to that of 2BF_2_-Me-Hex(b), indicating that the addition of boron ion in the last coordination site into 2BF_2_-Me-Hex(a) and 2BF_2_-Me-Hex(b) (Figure 4d). Interestingly, the other 3BF_2_-Me-Hex(b) shows a different peak pattern, which means that 3BF_2_-Me-Hex(b) has a different molecular configuration (Figure 4e). In the HR-MALDI-MS, corresponding molecular ion peaks of all boron complexes were observed (Figures S6–S10). Although the compounds Me-Por and Me-Oct reacted with BF_3_•OEt_2_ in the presence of TEA in CH_2_Cl_2_, boron complexes of Me-Por and Me-Oct could not be isolated. The reason is that the cavity space of compounds Me-Por and Me-Oct is insufficient to coordinate boron ions because of steric hindrances and less structural flexibilities.

**Figure 5:**
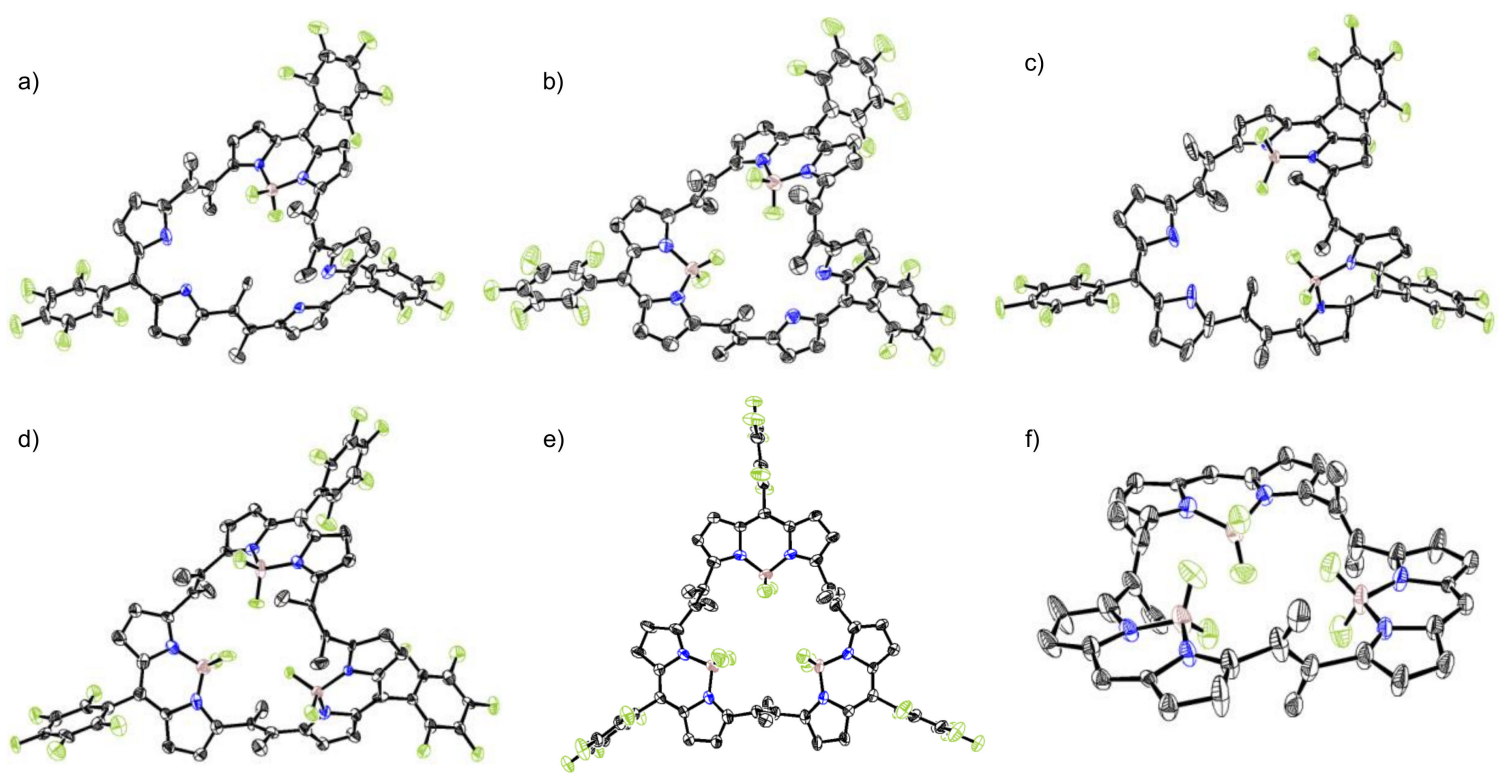
Crystal structures of (**a**) BF_2_-Me-Hex, (**b**) 2BF_2_-Me-Hex(a), (**c**) 2BF_2_-Me-Hex(b), (**d**) 3BF_2_-Me-Hex(a), and (**e**) 3BF_2_-Me-Hex(b), (**f**) side view of 3BF_2_-Me-Hex(b). The hydrogen atoms of five compounds, and pentafluorophenyl groups of (**f**) were omitted for clarity. The thermal ellipsoids are shown at 50% probability.

Molecular configurations of all boron complexes were determined by X-ray crystallography. BF_2_-Me-Hex, 2BF_2_-Me-Hex(a), 2BF_2_-Me-Hex(b), and 3BF_2_-Me-Hex(a) maintain distorted structures with *cis*/*cis*/*trans* geometry at vinylene bridges, which molecular frameworks are comparable to those of freebase Me-Hex (Figure 2 and Figure 5). The boron of BF_2_-Me-Hex is coordinated on the dipyrrin unit, which is located to the next to *cis*-vinylene bridge. This is consistent with the result of NMR (Figure 4a). The bis-boron complexes, 2BF_2_-Me-Hex(a) and 2BF_2_-Me-Hex(b), clearly show that positions of the second boron are different. The second boron of 2BF_2_-Me-Hex(a) is bonded to dipyrrin, which is sandwiched between *trans*-vinylene bridges, whereas the second boron of 2BF_2_-Me-Hex(b) is bonded to another site of dipyrrin. Triboron complex of 3BF_2_-Me-Hex(a) coordinates boron in all coordination sites and forms the distorted structure. In contrast, 3BF_2_-Me-Hex(b) has a different molecular structure with the *trans*-vinylene bridges and a co-planar structure.

**Figure 6:**
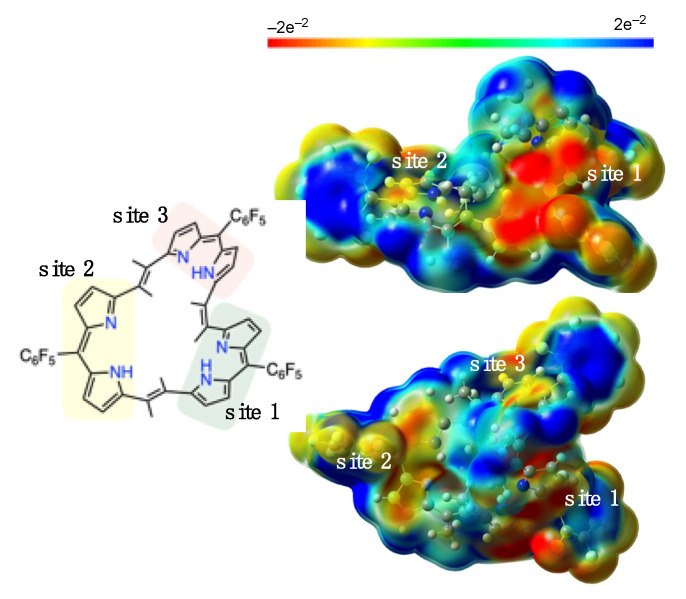
Electrostatic potential surface of Me-Hex. On top of the electrostatic potential surface, site 3 is located behind site 1.

To investigate the selectivity of boron complexation to Me-Hex, the electrostatic potential of Me-Hex was calculated by Gaussian 09 (Figure 6) [22]. The dipyrrin units are named as coordination site 1, site 2, and site 3, which are depicted in Figure 6. Site 1 shows the most negative potential (red-colored position) compared with the other dipyrrin sites. This means that site 1 dominantly produces the deprotonated dipyrrin species that can react with BF_3_•OEt_2_ to give BF_2_-Me-Hex.

**Figure 7:**
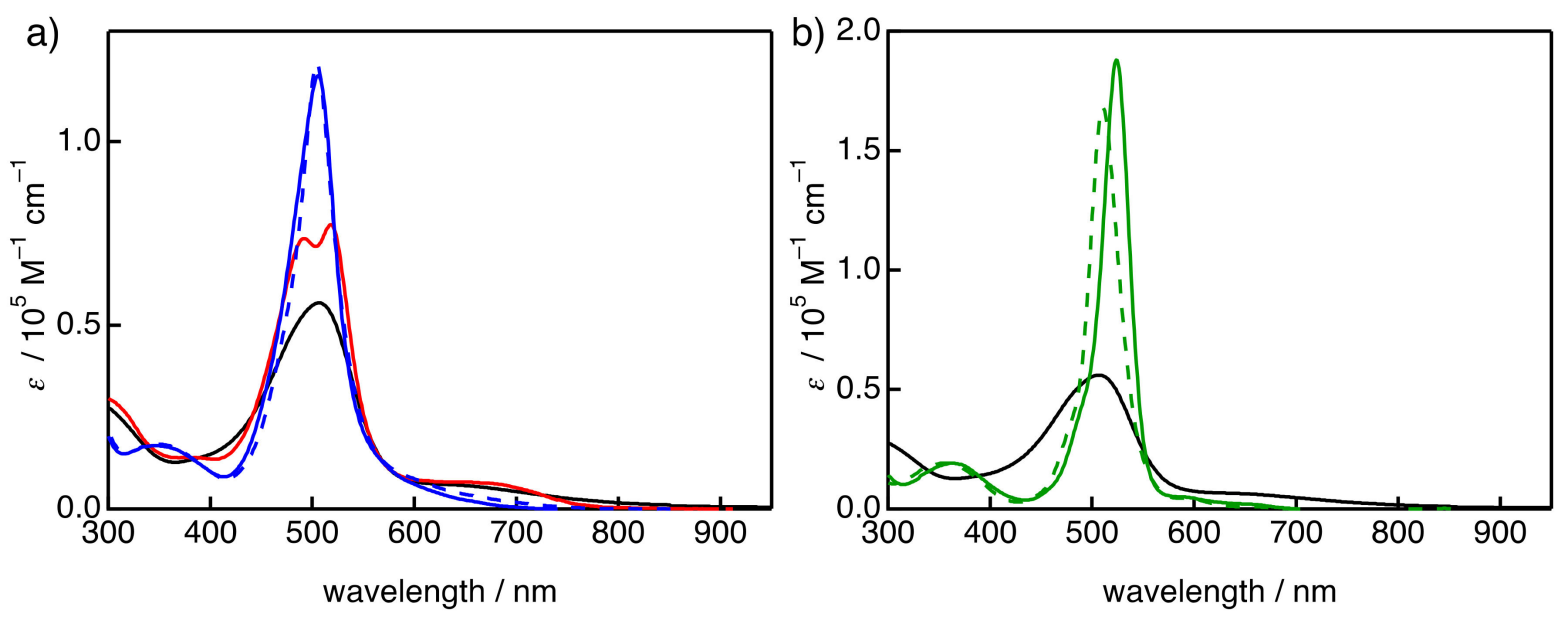
UV-vis absorption spectra of (**a**) Me-Hex (black), BF_2_-Me-Hex (red), 2BF_2_-Me-Hex(a) (solid blue), and 2BF_2_-Me-Hex(b) (dotted blue), and (**b**) Me-Hex (black), 3BF_2_-Me-Hex(a) (solid green), and 3BF_2_-Me-Hex(b) (dotted green) in CH_2_Cl_2_.

The absorption spectra of boron complexes are shown in Figure 7. All boron complexes show a strong absorption band around 450 to 550 nm, and a weak broad band around 600 to 850 nm. The strong bands of approximately 500 nm become sharpened and increase the absorption coefficient with an increase in the number of boron ions. Bis-boron complexes 2BF_2_-Me-Hex(a) and 2BF_2_-Me-Hex(b) exhibit similar absorption shapes, indicating that the position of boron ions does not strongly perturb the optical properties because they form similar molecular structures. In contrast, tri-boron complexes 3BF_2_-Me-Hex(a) and 3BF_2_-Me-Hex(b) exhibit slightly different absorption shapes, reflecting their different molecular structures. The emission spectra of all boron complexes were measured in CH_2_Cl_2_ (Figure S11). Freebase, mono, and bis-boron complexes have no fluorescence, even at low temperatures. In contrast, two tri-boron complexes exhibit a weak fluorescence in the red region (3BF_2_-Me-Hex(a): *λ*_em_ = 650 nm, *Φ*_F_ = 1.0%; 3BF_2_-Me-Hex(b): *λ*_em_ = 650 nm, *Φ*_F_ = 1.4%). These fluorescence quantum yields were obtained by comparison with *meso*-tetraphenylporphyrin (*Φ*_F_ = 0.12 in CH_2_Cl_2_) used as a standard [23,24].

**Figure 8:**
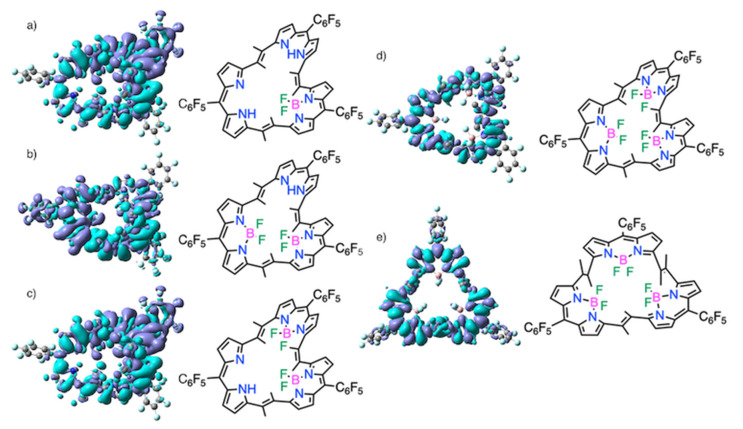
The electron density difference maps (EDDMs) of (**a**) BF_2_-Me-Hex, (**b**) 2BF_2_-Me-Hex(a), (**c**) 2BF_2_-Me-Hex(b), (**d**) 3BF_2_-Me-Hex(a), and (**e**) 3BF_2_-Me-Hex(b).

The optical properties of boron complexes were predicted by DFT and time dependent (TD)-DFT calculations at B3LYP/6-31G* level and cam-B3LYP/6-31G*// B3LYP/6-31G*, respectively. The HOMO of mono and bis-boron complexes of Me-Hex is located at the free-base dipyrrin unit, whereas their LUMO is mainly located at BODIPY units (Figures S12 and S13). In contrast, HOMO and LUMO of two tri-boron complexes 3BF_2_-Me-Hex(a) and 3BF_2_-Me-Hex(b) are equally delocalized on three BODIPY units. To deeper understand the optical properties of boron complexes, TD-DFT calculation was examined (Figure S14 and Table S1). The *S*_0_–*S*_1_ transitions of absorption of BF_2_-Me-Hex, 2BF_2_-Me-Hex(a), and 2BF_2_-Me-Hex(b) clearly exhibit an intramolecular charge transfer (ICT) transition from the free-base dipyrrin as the donor unit to the BODIPY unit as the acceptor [24,25,26]. In contrast, 3BF_2_-Me-Hex(a) and 3BF_2_-Me-Hex(b) exhibit a π-π transition. The electron density difference map (EDDM) represents the difference in electron densities upon electronic transitions (Figure 8) [27]. EDDMs of 3BF_2_-Me-Hex(a) and 3BF_2_-Me-Hex(b) are delocalized on the whole molecules, whereas the location of EDDMs for the lowest HOMO-LUMO transitions of BF_2_-Me-Hex, 2BF_2_-Me-Hex(a), and 2BF_2_-Me-Hex(b) is shifted from the free base dipyrrin units to the BODIPY units. The predicted ICT characteristics of BF_2_-Me-Hex, 2BF_2_-Me-Hex(a), and 2BF_2_-Me-Hex(b) are consistent with their non-emissive behavior in solution upon photoexcitation [25,26,27]. Generally, BODIPY compounds show strong emission. Actually, the *c*-BODIPY-3 is the emissive compound with high fluorescence quantum yield [14]. Unfortunately, although the lowest transition of Me-Hex is the π-π transition, 3BF_2_-Me-Hex(a) and 3BF_2_-Me-Hex(b) exhibit only weak fluorescence. There are two possible reasons. The first reason is that the *S*_0_-*S*_1_ transition of 3BF_2_-Me-Hex(a) and 3BF_2_-Me-Hex(b) is forbidden, inducing the low emissive property. The second reason is that molecular skeleton of Me-Hex is highly flexible, caused by the rotation of the vinylene bridges in the solution. It is advantageous to give high reactivity, but it induces the nonradiative deactivation. Therefore, to achieve the preparation of the high emissive cyclic BODIPYs, substituents on vinylene bridges of hexaphyrin(2.1.2.1.2.1) should be carefully chosen with the combination of reactivity and rigid molecular frameworks.

**Figure 9:**
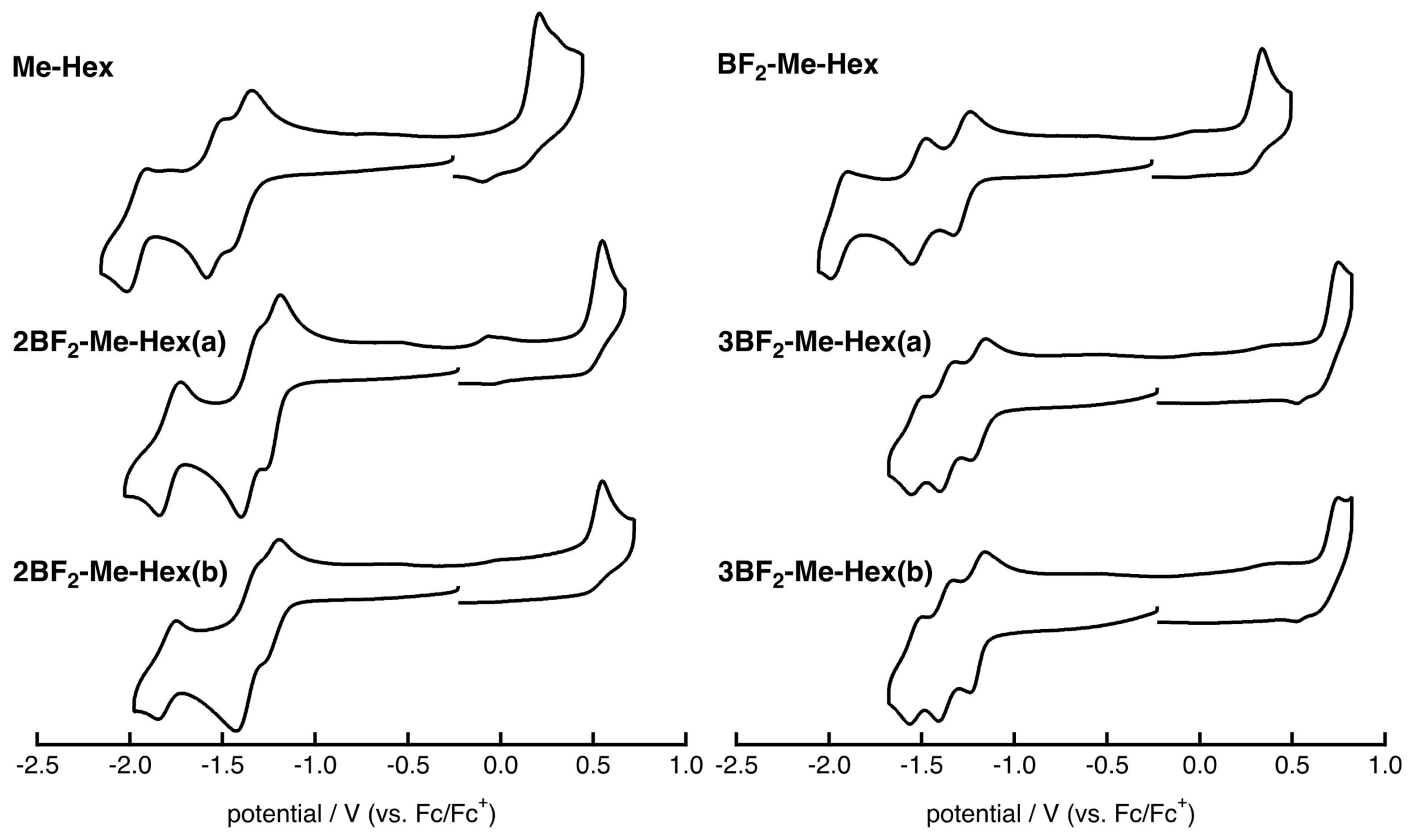
Cyclic voltammetry (CV) of Me-Hex and boron complexes in CH_2_Cl_2_ with 0.1 M TBAPF_6_. Scan rate is 0.1 V s^−1^.

The redox properties of BF_2_-Me-Hex, 2BF_2_-Me-Hex(a), 2BF_2_-Me-Hex(b), 3BF_2_-Me-Hex(a), and 3BF_2_-Me-Hex(b) were measured by cyclic voltammetry (CV) in CH_2_Cl_2_ with 0.1 M TBAPF_6_ as an electrolyte (Figure 9). The free base Me-Hex shows one irreversible oxidation and three reversible reduction potentials at 0.20, −1.40, −1.55, and −1.98 V (vs. Fc/Fc^+^). The cyclic BODIPYs also show similar redox properties. The oxidation and reduction potentials are gradually shifted positively because of the electron-withdrawing properties of boron–difluoride units [19].

## 3. Materials and Methods

### 3.1. General

^1^H NMR and ^19^F NMR spectra were recorded on a JNM-ECX 400 spectrometer (operating as 400 MHz for ^1^H and 376 MHz for ^19^F) using the residual solvent as the internal reference for ^1^H (*δ* = 7.26 ppm in CDCl_3_, *δ* = 5.32 ppm in CD_2_Cl_2_) and CF_3_COOH as the external reference for ^19^F (*δ* = −76.5 ppm). The ^1^H NMR and ^19^F NMR spectra of all compounds are shown in Figures S15–S32. HR-MALDI−TOF mass spectra were recorded on a Bruker Daltonics autoflex MALDI−TOF MS spectrometer. UV/Vis/NIR absorption spectra were measured using a JASCO UV/Vis/NIR Spectrophotometer V-670. CV measurements were conducted in a solution of 0.1 M TBAPF_6_ in dichloromethane at a scan rate of 0.1 V s^−1^ in an argon-filled cell. A glassy carbon electrode and a platinum wire were used as working and counter electrodes, respectively. An Ag/AgNO_3_ electrode was used as a reference electrode, which was normalized with the half-wave potential of ferrocene/ferrocenium (Fc/Fc^+^) redox couple. All solvents and chemicals were reagent grade quality, obtained commercially, and used without further purification, except as noted. For spectral measurements, spectral grade dichloromethane was purchased from Nacalai Tesque Co. Thin-layer chromatography (TLC), flush column chromatography, and gravity column chromatography were performed on Art. 5554 (Merck KGaA, Darmstadt, Germany), Silica Gel 60 (Merck KGaA, Darmstadt, Germany), and Silica Gel 60N (Kanto Chemical Co., Tokyo, Japan), respectively.

### 3.2. X-ray Analysis

All the single crystals except Me-Oct were obtained by the solvent diffusion method. The solvents are described in the Tables S2–S9 in the supporting information. Single crystals of Me-Oct were obtained by recrystallization from toluene. X-ray crystallographic data for BF_2_-Me-Hex, 2BF_2_-Me-Hex(b), and 3BF_2_-Me-Hex(b) were recorded at 90 K using a BRUKER-APEXII X-Ray diffractometer using a Mo-Kα radiation equipped with a large area CCD detector and X-ray crystallographic data for Me-Por, Me-Hex, Me-Oct, 2BF_2_-Me-Hex(a), and 3BF_2_-Me-Hex(a) were recorded at 103 K on a Rigaku R-AXIS RAPID/S imaging plate diffractometer using Mo-Kα radiation. The structures were solved by direct methods and refined on *F^2^* by full−matrix least−squares using the CrystalClear and SHELXS−97 and SHELXT−2014/5 programs. CCDC: 1830225, 1830228, 1830229, 1830230, 1830231, 1848201, 1830232, and 1830233 contain the supplementary crystallographic data for Me-Por, Me-Hex, Me-Oct, BF_2_-Me-Hex, 2BF_2_-Me-Hex(a), 2BF_2_-Me-Hex(b), 3BF_2_-Me-Hex(a), and 3BF_2_-Me-Hex(b), respectively. These data can be obtained free of charge from the Cambridge Crystallographic Data Centre via www.ccdc.cam.ac.uk/data_request/cif.

### 3.3. Theoretical Calculations

All density functional theory calculations were achieved with the Gaussian 09 program package. The geometry was optimized at the Becke’s three-parameter hybrid functional combined with the Lee−Yang−Parr correlation functional abbreviated as the B3LYP level of density functional theory with the 6-31G(d) basis set.

### 3.4. Synthesis of Dimethyl-Dipyrrolylethane (E/Z-1)

TiCl_4_ (3.5 mL) was added dropwise to a solution of Zn powder (2.73 g) and CuCl (273 mg) in THF (75 mL) at 0 °C under argon. The reaction mixture was heated at reflux for 2 h. 2-Acetylpyrrole (600 mg, 5.5 mmol) in THF (10 mL) was added dropwise to the reaction mixture, and then the solution was heated at reflux for another 2 h. The reaction was carefully quenched with aqueous sodium acetate and the resulting mixture was extracted with CH_2_Cl_2_. The organic layer was washed with water and brine, and dried over anhydrous Na_2_SO_4_. After removal of the solvent, the crude product was purified by silica gel column chromatography (CH_2_Cl_2_/hexane = 3/2) to give *Z*-1 in 23% (120 mg, 0.6 mmol) and *E*-1 in 10% (51 mg, 0.3 mmol). *Z*-1: ^1^H NMR (400 MHz, CDCl_3_, 298 K) *δ* = 7.74 (brs, 2H), 6.60 (m, 2H, pyrrole), 6.18–6.17 (m, 4H, pyrrole), 2.12 (s, 6H, methyl) ppm. *E*-1: ^1^H NMR (400 MHz, CDCl_3_, 298 K) *δ* = 8.17 (brs, 2H, NH), 6.81–6.80 (m, 2H, pyrrole), 6.28–6.26 (m, 2H, pyrrole), 6.24–6.23 (m, 2H, pyrrole), 2.22 (s, 6H, methyl) ppm.

### 3.5. Synthesis of Me-Por, Me-Hex, and Me-Oct

To a solution of *E/Z-*1 (186 mg, 1.0 mmol) and pentafluorobenzaldehyde (196 mg, 1.0 mmol) in CH_2_Cl_2_ (150 mL) was added BF_3_•Et_2_O (4.3 mg, 0.03 mmol) under argon. After stirring for 2 h at room temperature, DDQ (227 mg, 1.0 mmol) was added to the reaction mixture, which was stirred for 1 h. After removal of the solvent, the residue was purified by alumina chromatography (hexane/CH_2_Cl_2_ = 5/1) and silica gel column chromatography (CH_2_Cl_2_). The first eluted yellow fraction was evaporated to give Me-Por in 1% (3.5 mg, 0.0048 mmol) as a deep purple solid. The second red fraction was evaporated to give Me-Hex in 6% (21.5 mg, 0.02 mmol) as a dark red solid, and the third green fraction was evaporated to give Me-Oct in 3% (10.5 mg, 0.0075 mmol) as a dark red solid.

Me-Por: ^1^H NMR (CDCl_3_, 400 MHz, 298 K) *δ* = 11.82 (brs, 2H), 6.23 (d, *J* = 4 Hz, 4H), 6.12 (d, *J* = 4 Hz, 4H), 2.16 (s, 12H) ppm. ^19^F NMR (CDCl_3_, 376 MHz, CF_3_COOH) *δ* = −152.98–−153.10 (t, 2F), −161.26–−161.40 (m, 2F), −161.49–−161.62 (m, 2F) ppm. HR-MALDI-MS: Calculated for C_38_H_22_F_10_N_4_: 724.1685 [M^+^], Found: 724.1683. UV-vis-NIR (in CH_2_Cl_2_) *λ* [nm] (*ε*[M^−1^cm^−1^]): 431 (58,000) 507 (sh.) (4300).

Me-Hex: ^1^H NMR (CDCl_3_, 400 MHz, 298 K) δ = 14.14 (brs, 1H), 13.62 (brs, 1H), 13.25 (brs, 1H), 6.75 (d, *J* = 4 Hz, 1H), 6.66 (d, *J* = 4 Hz, 1H), 6.59 (d, *J* = 4 Hz, 1H), 6.51 (d, *J* = 4 Hz, 1H), 6.48 (d, *J* = 4 Hz, 1H), 6.45 (d, *J* = 4 Hz, 1H), 6.43 (d, *J* = 4 Hz, 1H), 6.39 (d, *J* = 4 Hz, 1H), 6.36 (d, *J* = 4 Hz, 1H), 6.03 (d, *J* = 4 Hz, 1H), 5.96 (d, *J* = 4 Hz, 1H), 5.56 (d, *J* = 4 Hz, 1H), 2.71 (s, 3H), 2.57 (s, 3H), 2.45 (s, 3H), 2.40 (s, 3H), 2.29 (s, 3H), 1.95 (s, 3H) ppm. ^19^F NMR (CDCl_3_, 376 MHz, CF_3_COOH) *δ* = −136.84–−138.37 (m, 6F), −152.23–−153.37 (m, 3F), −160.92–−161.38 (m, 6F) ppm. HR-MALDI-MS: Calculated for C_57_H_33_F_15_N_6_: 1086.2527 [M^+^], Found: 1086.2522. UV-vis-NIR (in CH_2_Cl_2_) *λ* [nm] (*ε* [M^−1^ cm^−1^]): 506 (84,000), 645 (sh) (9700).

Me-Oct: ^1^H NMR (400 MHz, CDCl_3_, 298 K) *δ* = 12.49 (brs, 4H), 6.43 (s, 4H), 6.33 (d, *J* = 4 Hz, 4H), 6.23 (s, 8H), 2.33 (s, 12H), 2.29 (s, 12H) ppm. ^19^F NMR (376 MHz, CDCl_3_): *δ* = −137.84–−137.90 (m, 8F), −152.78–−152.90 (m, 4F), −161.15–−161.26 (m, 8F) ppm. HR-MALDI-MS: Calculated for C_76_H_44_F_20_N_8_: 1448.3370 [M^+^], Found: 1448.3370. UV-vis-NIR (in CH_2_Cl_2_) *λ* [nm] (*ε* [M^−1^ cm^−1^]): 438 (43,000), 570 nm (72,000).

### 3.6. Synthesis of Boron Complexes of Me-Hex

Method A: To a solution of Me-Hex (11 mg, 0.01 mmol) in CH_2_Cl_2_ (20 mL) and triethylamine (TEA) (40 mg, 0.4 mmol) was added BF_3_•Et_2_O (113 mg, 0.8 mmol) under argon. After stirring at room temperature for 12 h, the reaction mixture was extracted with CH_2_Cl_2_. The organic phase was washed with aqueous NaHCO_3_, water and brine, and dried over Na_2_SO_4_. After removal of the solvent, the crude product was purified by silica gel column chromatography (hexane/CH_2_Cl_2_ = 3/1) to give BF_2_- Me-Hex (7 mg, 0.006 mmol) in 60% yield.

Method B: To a solution of Me-Hex (11 mg, 0.01 mmol) in CH_2_Cl_2_ (20 mL) and triethylamine (TEA) (40 mg, 0.4 mmol) was added BF_3_•Et_2_O (169 mg, 1.2 mmol) under argon. After stirring at reflux for 12 h, the reaction mixture was extracted with CH_2_Cl_2_. The organic phase was washed with aqueous NaHCO_3_, water and brine, and dried over Na_2_SO_4_. After removal of the solvent, the resulting crude product was purified by silica gel column chromatography (hexane/CH_2_Cl_2_ = 3/2) to give 2BF_2_-Me-Hex(a) (2.4 mg, 0.002 mmol) in 20% and 2BF_2_-Me-Hex(b) (3.4 mg, 0.0028 mmol) in 28%.

Method C: To a solution of Me-Hex (10.8 mg, 0.01 mmol) in toluene (20 mL) and triethylamine (TEA) (40 mg, 0.4 mmol) was added BF_3_•Et_2_O (169 mg, 1.2 mmol) under argon. After stirring at 80 °C for 12 h, the reaction mixture was extracted with CH_2_Cl_2_. The organic phase was washed with aqueous NaHCO_3_, water, and brine, and dried over Na_2_SO_4_. After removal of the solvent, the resulting crude product was purified by silica gel column chromatography (hexane/CH_2_Cl_2_ = 1/1) to give 3BF_2_-Me-Hex(a) (0.63 mg, 0.0005 mmol) in 5% and 3BF_2_-Me-Hex(a) (0.25 mg, 0.0002 mmol) in 2%, 2BF_2_-Me-Hex(a) in 20% (2.4 mg, 0.002 mmol), and 2BF_2_-Me-Hex(b) in 28% (3.4 mg, 0.0028 mmol).

BF_2_-Me-Hex: ^1^H NMR (400 MHz, CDCl_3_, 298 K) *δ* = 13.26 (brs, 1H), 13.07 (brs, 1H), 6.75 (d, *J* = 4 Hz, 2H), 6.69 (d, *J* = 4 Hz, 1H), 6.65 (d, *J* = 4 Hz, 1H), 6.60 − 6.59 (m, *J* = 4 Hz, 2H), 6.54 (d, *J* = 4 Hz, 1H), 6.49 (d, *J* = 4 Hz, 1H), 6.45 (d, *J* = 4 Hz, 1H), 6.28 (d, *J* = 4 Hz, 1H), 6.24 (d, *J* = 4 Hz, 1H), 6.22 (d, *J* = 4 Hz, 1H), 2.73 (s, 3H), 2.47 (s, 3H), 2.42 (s, 3H), 2.21 (s, 3H), 2.18 (s, 3H), 1.96 (s, 3H) ppm. ^19^F NMR (376 MHz, CDCl_3_): *δ* = −135.60 (m, 1F), −136.56 (m, 2F), −137.68 (m, 1F), −137.95 (m, 2F), −141.50 (brs, 2F), −150.02 (t, *J* = 41 Hz, 1F), −152.46 (t, *J* = 42 Hz, 1F), −152.69 (t, *J* = 41 Hz, 1F), −159.67 (m, 2F), −160.78 (m, 2), −161.15 (m, 2F) ppm. HR-MALDI-MS: Calculated for C_57_H_32_BF_17_N_6_ = 1134.2510 [M]^+^, Found: 1134.2505. UV-vis-NIR (in CH_2_Cl_2_) *λ* [nm] (*ε* [M^−1^cm^−1^]): 492 (54,000), 518 (58,000).

2BF_2_-Me-Hex(a): ^1^H NMR (400 MHz, CDCl_3_, 298 K) *δ* = 6.76 (d, *J* = 4 Hz, 1H), 6.71 (d, *J* = 4 Hz, 1H), 6.66 (d, *J* = 4 Hz, 1H), 6.62 (d, *J* = 4 Hz, 1H), 6.47 (m, 2H), 6.41 (m, 2H), 6.38 (d, *J* = 4 Hz, 1H), 6.34 (d, *J* = 4 Hz, 1H), 6.31 (d, *J* = 4 Hz, 1H), 6.23 (d, *J* = 4 Hz, 1H), 2.66 (s, 3H), 2.26 (s, 3H), 2.24 (s, 3H), 2.15(s, 3H), 2.14 (s, 3H), 1.95 (s, 3H) ppm. ^19^F NMR (376 MHz, CDCl_3_): *δ* = −135.14 (m, 1F), −136.01 (m, 1F), −136.45 (m, 2F), −136.85 (m, 1F), −138.06 (m, 1F), −138.07 (brs, 1F), −138.72 (brs, 1F), −144.69 (brs, 1F), −146.38 (brs, 1F), −150.01 (t, *J* = 42 Hz, 1F), −150.20 (t, *J* = 42 Hz, 1F), −152.86 (t, *J* = 42 Hz, 1F), −159.61 (m, 3F), −159.95 (m, 1F), −161.33 (m, 2F) ppm. HR-MALDI-MS: Calculated for C_57_H_31_B_2_F_19_N_6_Na = 1205.2391 [M+Na]^+^, Found: 1205.2391. UV-vis-NIR (in CH_2_Cl_2_) λ [nm] (*ε* [M^−1^cm^−1^]): 510 (11,4000).

2BF_2_-Me-Hex(b): ^1^H NMR (400 MHz, CDCl_3_, 298 K) *δ* = 6.69 (d, *J* = 4 Hz, 2H), 6.61 (d, *J* = 4 Hz, 2H), 6.53 (d, *J* = 4 Hz, 4H), 6.36 (d, *J* = 4 Hz, 2H), 6.34 (d, *J* = 4 Hz, 2H), 2.41 (s, 6H), 2.26 (s, 6H), 2.06 (s, 6H) ppm. ^19^F NMR (376 MHz, CDCl_3_, 213 K): *δ* = −133.07 (brs, 1F), −135.15 (m, 1F), −135.02 (m, 1F), −135.53 (m, 1F), −136.13 (m, 1F), −137.12 (m, 1F), −137.90 (m, 1F), −139.10 (brs, 1F), −143.15 (brs, 1F), −149.11 (t, *J* = 42 Hz, 1F), −149.83 (brs, 1F), −151.55 (t, *J* = 42 Hz, 1F), −158.93 (m, 3F), −159.83 (m, 1F), −160.16 (m, 2F) ppm. HR-MALDI-MS: Calculated for C_57_H_31_B_2_F_19_N_6_Na = 1205.2391 [M+Na]^+^, Found: 1205.2397. UV-vis-NIR (in CH_2_Cl_2_) *λ* [nm] (*ε* [M^−1^ cm^−1^]): 500 (12,3000).

3BF_2_-Me-Hex(a): ^1^H NMR (400 MHz, CDCl_3_, 298 K) *δ* = 6.70 (d, *J* = 4 Hz, 2H), 6.63 (d, *J* = 4 Hz, 2H), 6.50 (d, *J* = 4 Hz, 2H), 6.45 (d, *J* = 4 Hz, 2H), 6.43 (d, *J* = 4 Hz, 2H), 6.29 (d, *J* = 4 Hz, 2H), 2.32 (s, 3H), 2.29 (s, 3H), 2.22 (s, 3H) ppm. ^19^F NMR (376 MHz, CDCl_3_): *δ* = −128.81 (brs, 2F), −132.02 (brs, 1F), −135.13 (m, 2F), −136.34 (m, 1F), −136.71 (m, 3F), −149.10 (brs, 1F), −150.09 (t, *J* = 41 Hz, 1F), −150.32 (t, *J* = 43 Hz, 2F), −151.50 (brs, 1F), −159.88 (m, 6F) ppm. HR-MALDI-MS: Calculated for C_57_H_30_B_3_F_21_N_6_Na = 1253.2393 [M+Na]^+^, Found: 1253.2399. UV-vis-NIR (in CH_2_Cl_2_) *λ* [nm] (*ε* [M^−1^cm^−1^]): 525 nm (18,7000).

3BF_2_-Me-Hex(b): ^1^H NMR (400 MHz, CDCl_3,_ 298 K) *δ* = 6.76 − 6.73 (m, 6H), 6.36 − 6.31 (m, 6H), 2.13 (s, 6H), 2.11 (s, 6H,), 2.03 (s, 3H), 2.02 (s, 3H) ppm. ^19^F NMR (376 MHz, CDCl_3_): *δ* = −136.37 (m, 6F), −139.86 (m, 2F), −143.61 (brs, 2F), −147.17 (brs, 2F), −150.10 (m, 3F), −159.64 (m, 6F) ppm. HR-MALDI-MS: Calculated for C_57_H_30_B_3_F_21_N_6_Na = 1253.2393 [M+Na]^+^, Found: 1253.2393. UV-vis-NIR (in CH_2_Cl_2_) *λ* [nm] (*ε* [M^−1^ cm^−1^]): 512 (16,7000).

## 4. Conclusions

Five cyclic BODIPYs were synthesized from dimethyl-vinylene bridged hexaphyrin Me-Hex as effective expanded porphyrin-like ligand. The BF_2_-Me-Hex, 2BF_2_-Me-Hex(a), 2BF_2_-Me-Hex(b), and 3BF_2_-Me-Hex(a) form the distorted structure reflecting the molecular structure of Me-Hex. Alternatively, 3BF_2_-Me-Hex(b) forms the co-planar structure, meaning that the transformation of the molecular skeleton of Me-Hex occurs during boron complexation. Optical property of cyclic BODIPYs affects their molecular structures. The macrocycles BF_2_-Me-Hex, 2BF_2_-Me-Hex(a) and 2BF_2_-Me-Hex(b) having both the dipyrrin and BODIPY units exhibit no fluorescence because of ICT characteristics. In contrast, cyclic BODIPY trimers that consist only of BODIPY units show weak fluorescence. In addition, we experimentally revealed that the number of boron ions on cyclic BODIPYs is an important factor for the determination of their optical and electronic properties. Currently, we have been developing the chemistry of cyclic dipyrrins that are promising ligands for preparing cyclic BODIPY, which will take us to the next step for research on optical and electronic materials.

## Supplementary Materials

The followings are available online at https://www.mdpi.com/1422-0067/21/21/8041/s1. Figures S1–S3: HRMS spectra of Me-Por. Me-Hex and Me-Oct; Figure S4: Energy diagram of Me-Hex and Me-Oct; Figure S5: Molecular orbitals of Me-Hex and Me-Oct; Figures S6–S10: HRMS spectra of BF_2_-Me-Hex, 2BF_2_-Me-Hex(a), 2BF_2_-Me-Hex(b), 3BF_2_-Me-Hex(a), and 3BF_2_-Me-Hex(b); Figure S11: Fluorescence spectra of 3BF_2_-Me-Hex(a), and 3BF_2_-Me-Hex(b); Figure S12: Energy diagram of BF_2_-Me-Hex, 2BF_2_-Me-Hex(a), 2BF_2_-Me-Hex(b), 3BF_2_-Me-Hex(a), and 3BF_2_-Me-Hex(b); Figure S13: Molecular orbitals of BF_2_-Me-Hex, 2BF_2_-Me-Hex(a), 2BF_2_-Me-Hex(b), 3BF_2_-Me-Hex(a), and 3BF_2_-Me-Hex(b); Figure S14 and Table S1: Simulated absorption spectra BF_2_-Me-Hex, 2BF_2_-Me-Hex(a), 2BF_2_-Me-Hex(b), 3BF_2_-Me-Hex(a), and 3BF_2_-Me-Hex(b); Figures S15–S32: ^1^H and ^19^F NMR spectra; Tables S2–S9: crystal data.
