# Supplementary material for: Vinylene-Bridged Cyclic Dipyrrin and BODIPY Trimers
Source: Int J Mol Sci. 2020 Oct 28;21(21):8041. doi: 10.3390/ijms21218041 (PMC7662884; doi:10.3390/ijms21218041)
Supplement: Supplementary file 1 [file ijms-21-08041-s001.pdf]

Supporting Information

Vinylene-Bridged Cyclic Dipyrrins and BODIPY Trimers

Songlin Xue[1], Daiki Kuzuhara[*2], Naoki Aratani[3] and Hiroko Yamada[*3]

[1]School of Chemistry and Chemical Engineering, Jiangsu University, 301 Xuefu Road, Zhenjiang 212013, China

[2]Faculty of Science and Engineering, Iwate University, 4-3-5 Ueda, Morioka, Iwate 020-8551, Japan; kuzuhara@iwate-u.ac.jp

[3]Division of Materials Science, Nara Institute of Science and Technology, 8916-5 Takayama-cho, Ikoma, Nara 630-0192, Japan; hyamada@ms.naist.jp

Supporting Figures

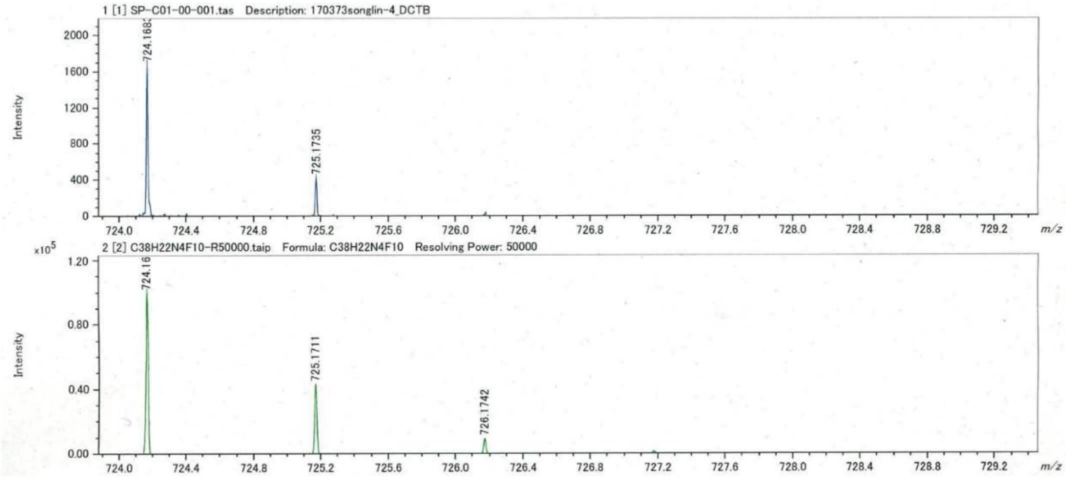

Figure S1. Observed (top) and simulated (bottom) HR-MALDI-MS spectra of **Me-Por**.

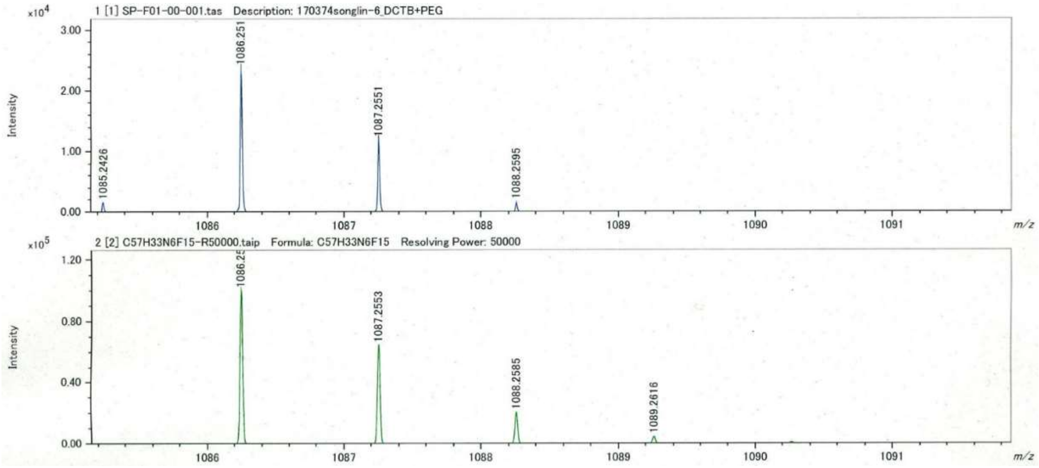

Figure S2. Observed (top) and simulated (bottom) HR-MALDI-MS spectra of **Me-Hex**.

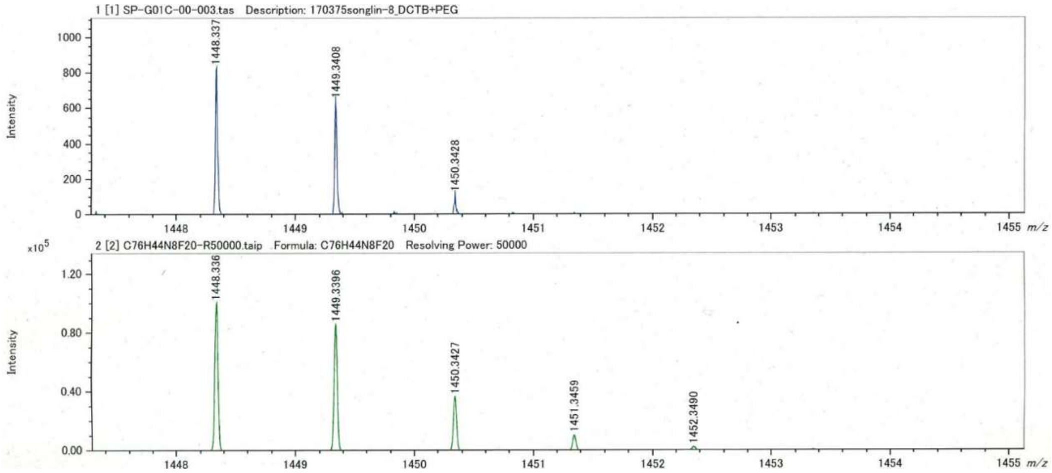

Figure S3. Observed (top) and simulated (bottom) HR-MALDI-MS spectra of **Me-Oct**.

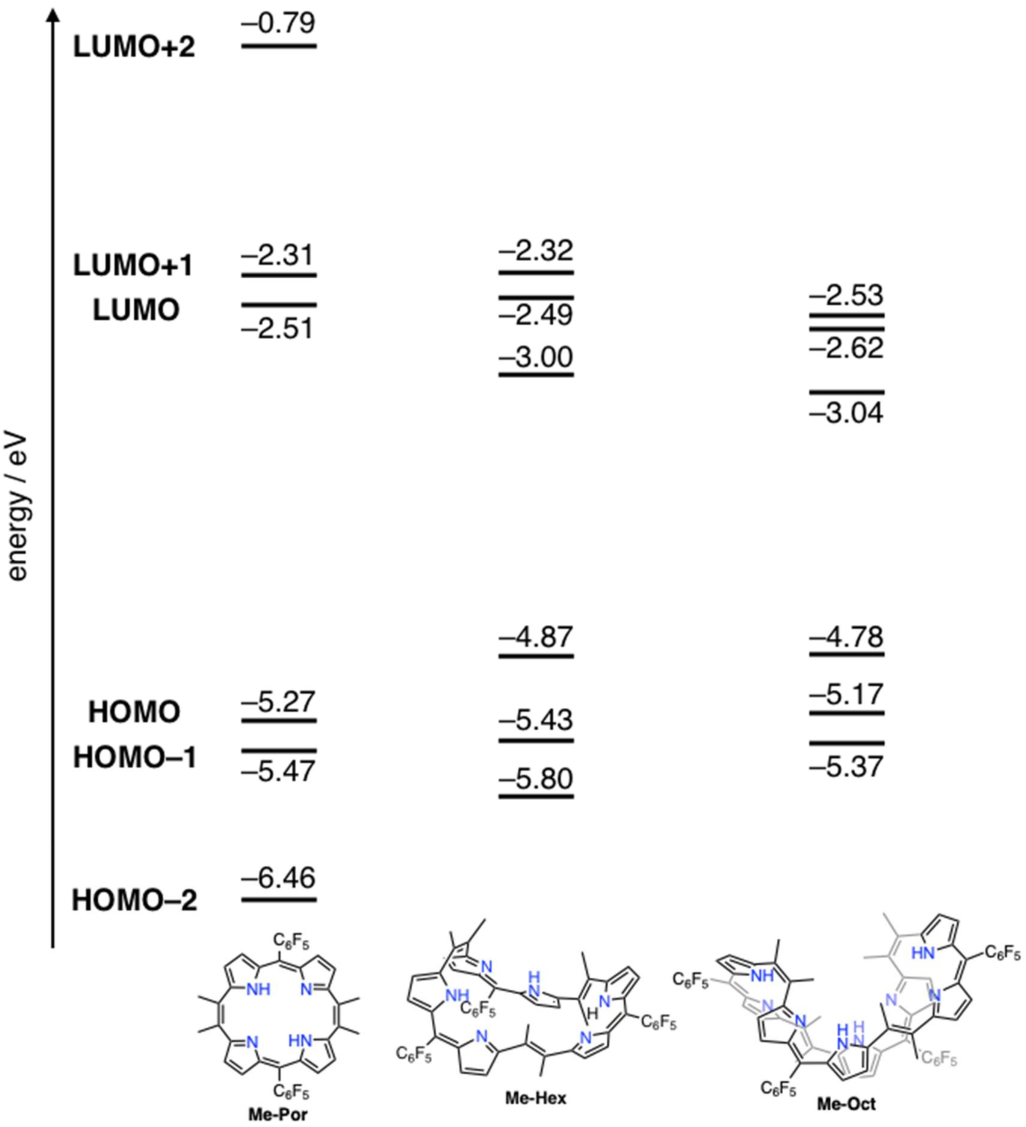

Figure S4. Energy diagram of **Me-Por**, **Me-Hex**, and **Me-Oct** calculated by B3LYP/6-31G* level.

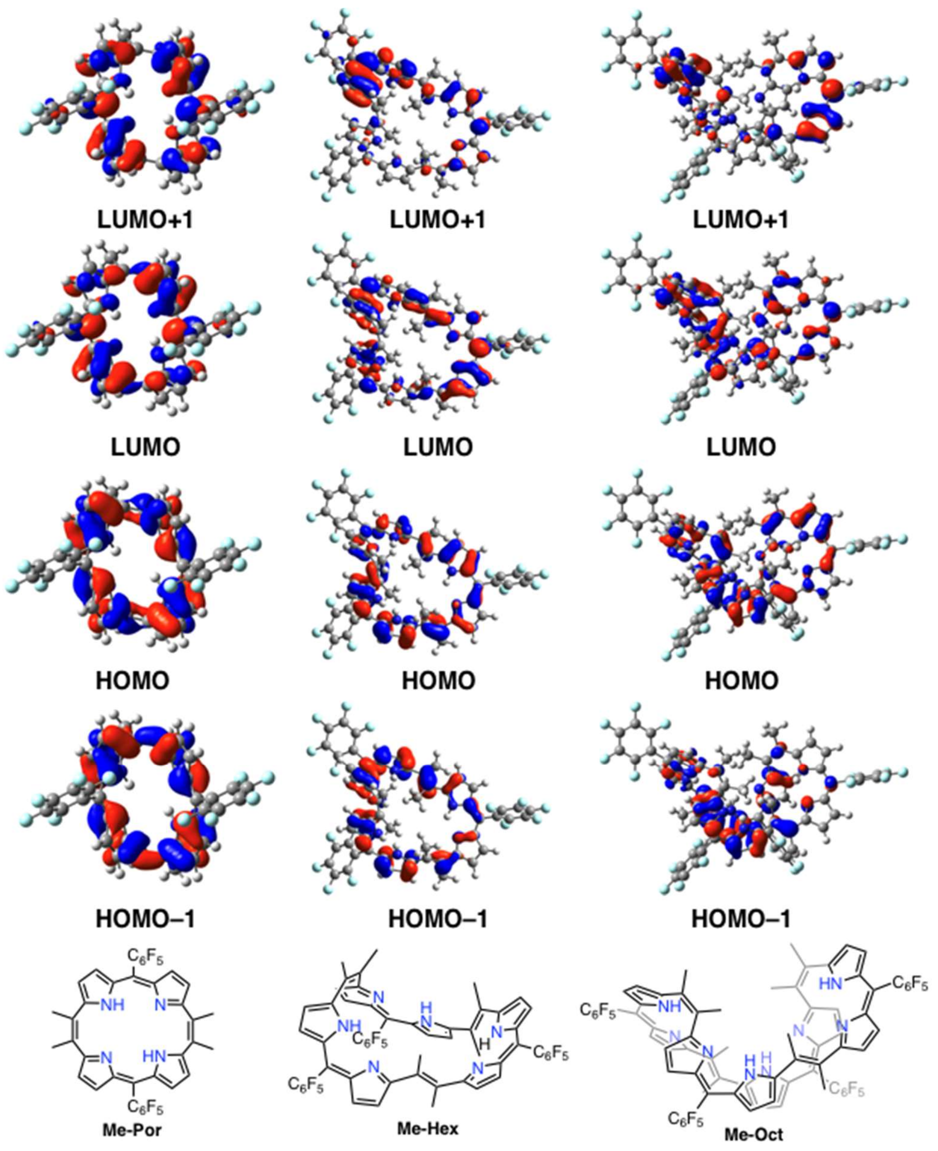

Figure S5. Kohn-Sham molecular orbitals of **Me-Por**, **Me-Hex**, and **Me-Oct** calculated by B3LYP/6-31G* level.

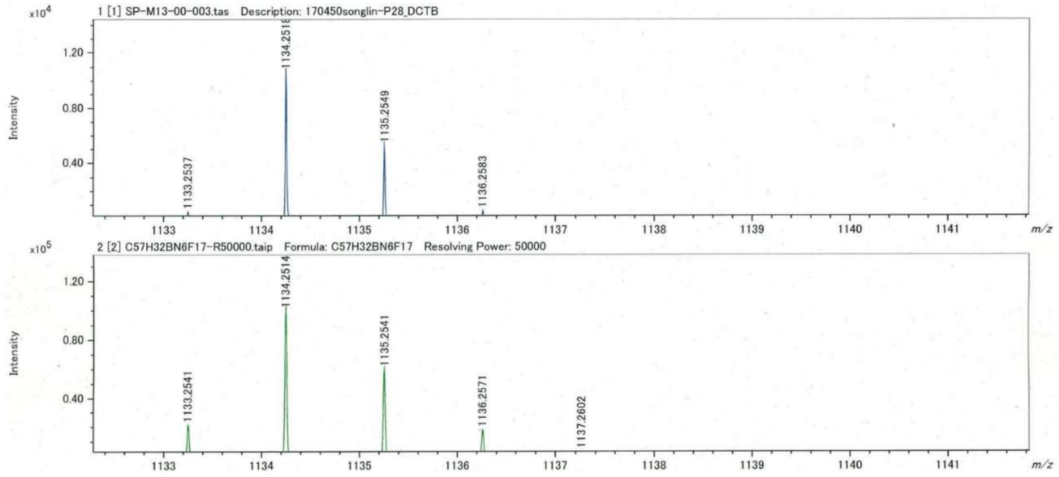

Figure S6. Observed (top) and simulated (bottom) HR-MALDI-MS spectra of **BF_2-Me-Hex**.

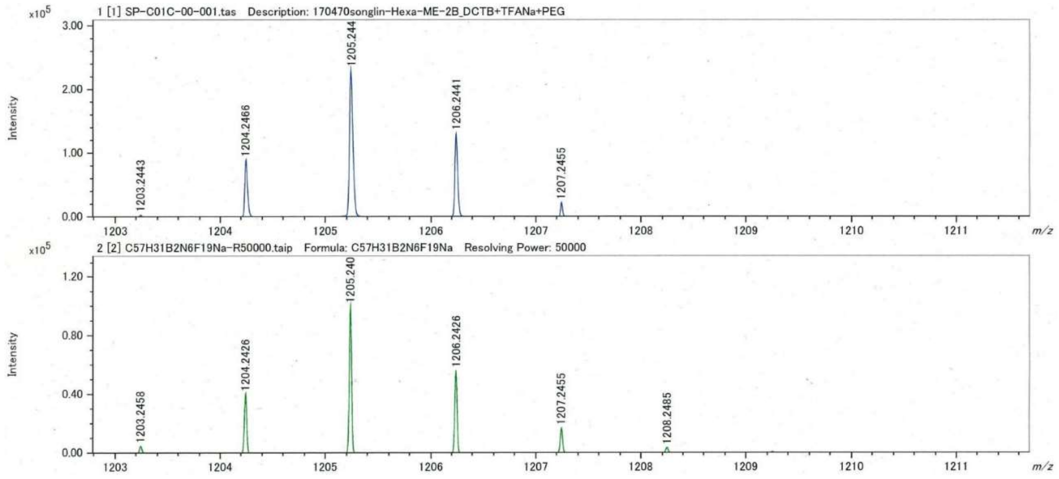

Figure S7. Observed (top) and simulated (bottom) HR-MALDI-MS spectra of **2BF_2-Me-Hex(a)**.

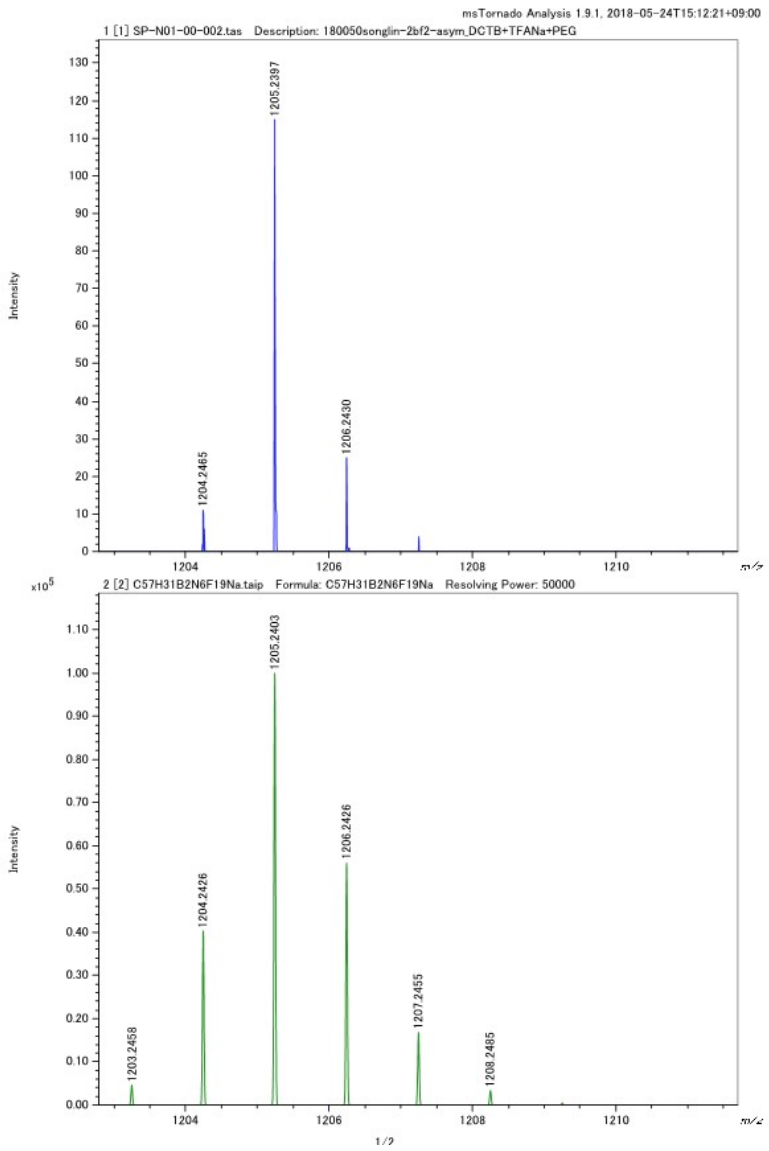

Figure S8. Observed (top) and simulated (bottom) HR-MALDI-MS spectra of **$2BF_2$-Me-Hex(b)**.

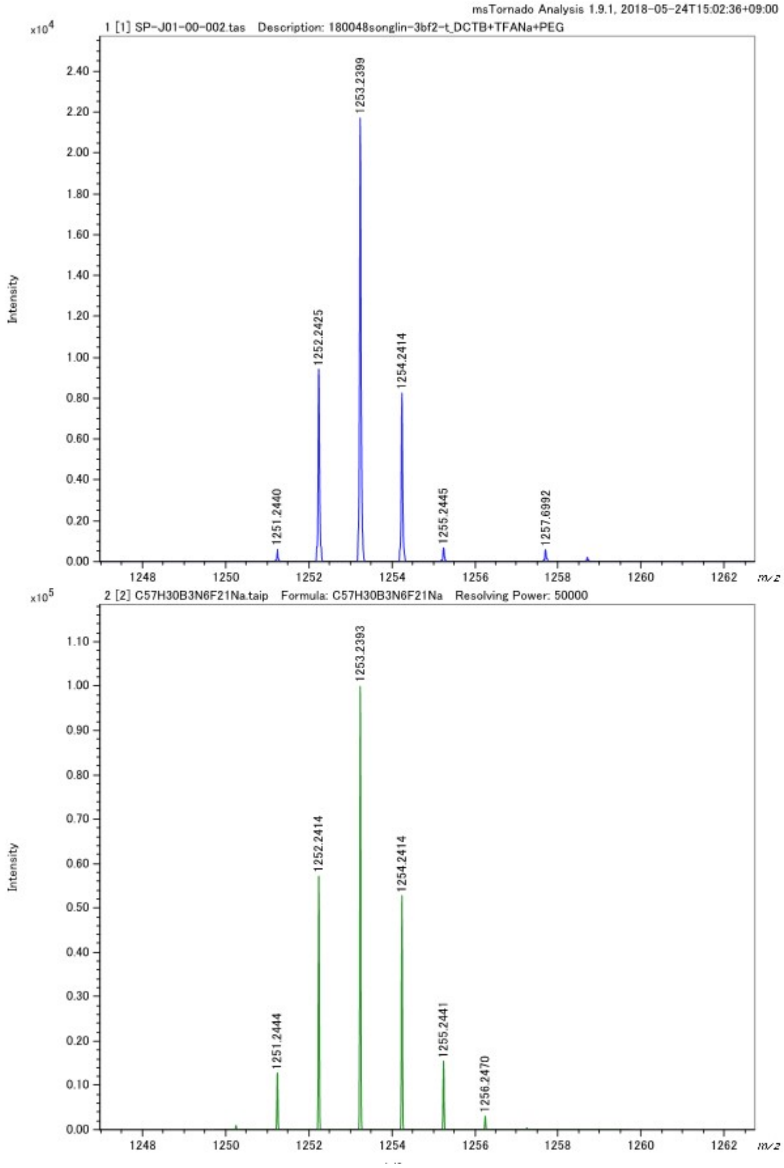

Figure S9. Observed (top) and simulated (bottom) HR-MALDI-MS spectra of **3BF$_2$-Me-Hex(a)**.

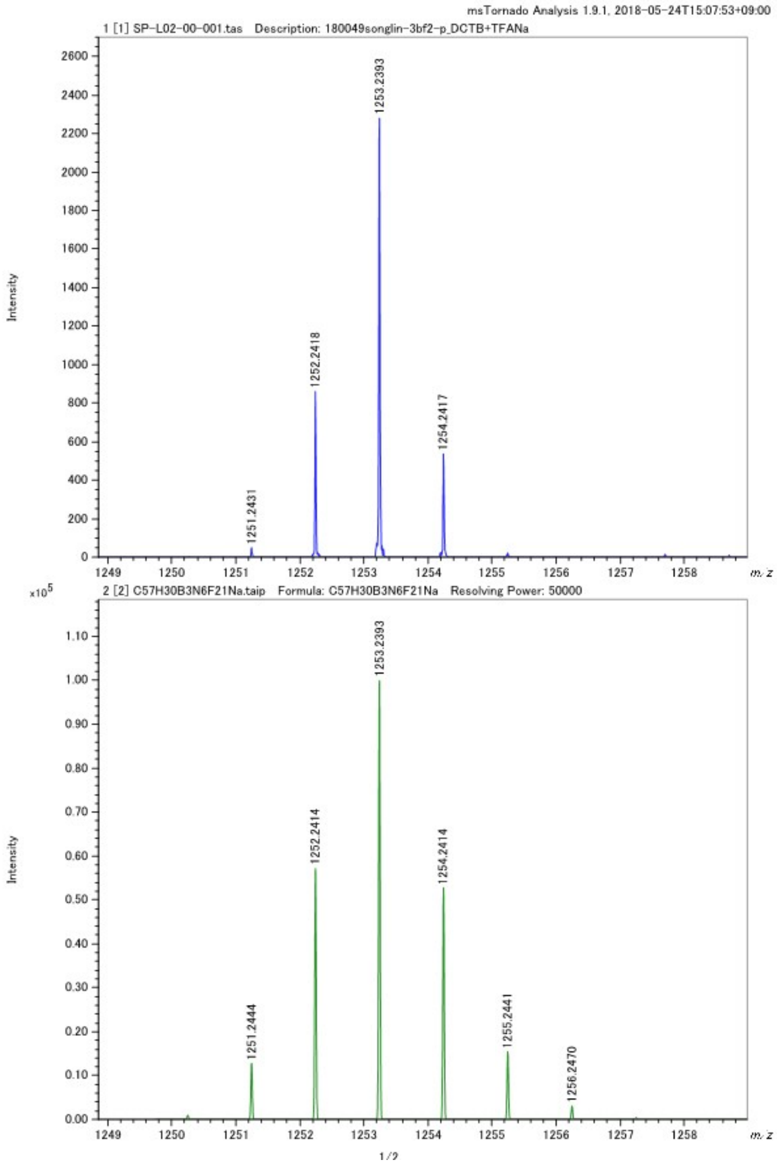

Figure S10. Observed (top) and simulated (bottom) HR-MALDI-MS spectra of **3BF$_2$-Me-Hex(b)**.

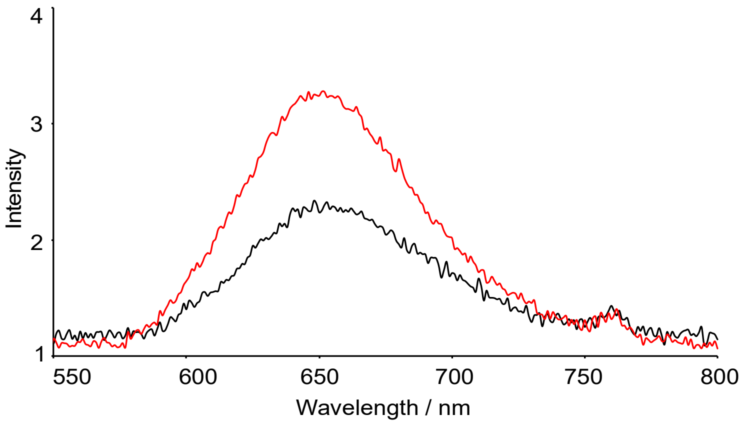

Figure S11. Fluorescence spectra of **3BF$_2$-Me-Hex(a)** (black line) and **3BF$_2$-Me-Hex(b)** (red line) in CH_2Cl_2. The excitation wavelength of two complexes were set at 508 nm.

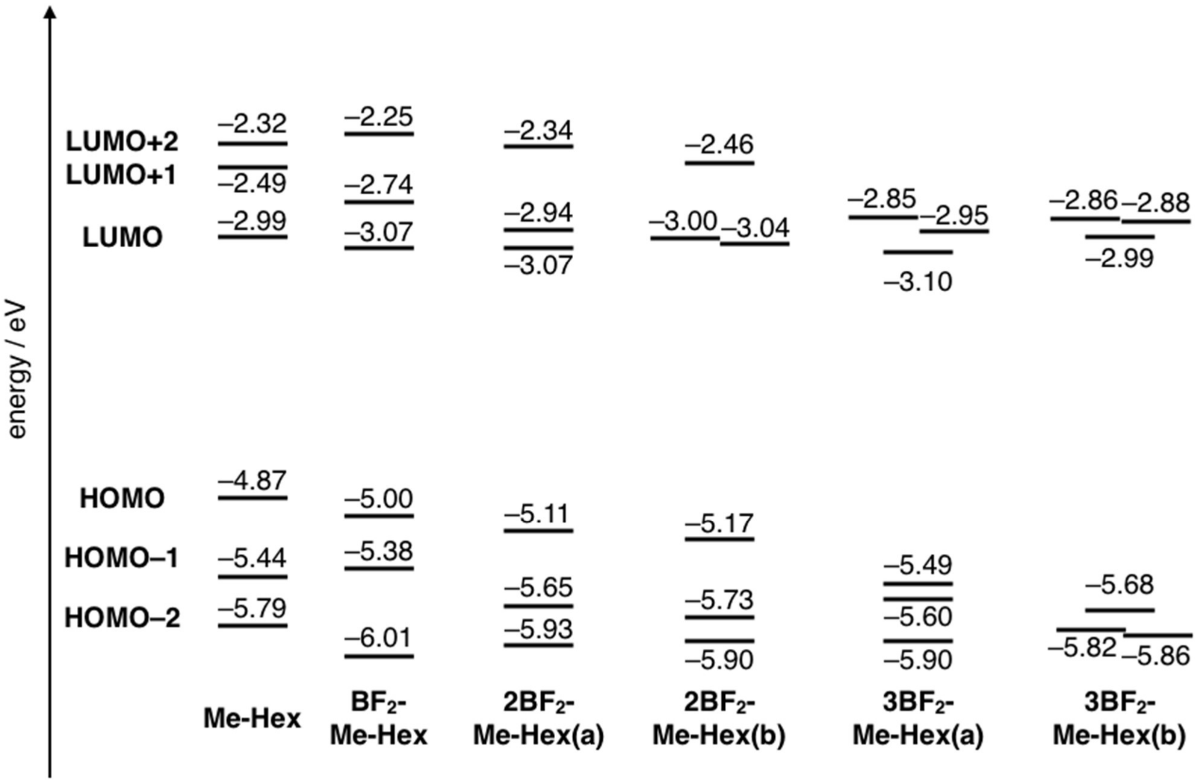

Figure S12. Energy diagram of **Me-Hex** and boron complexes calculated at the B3LYP/6-31G* level of theory.

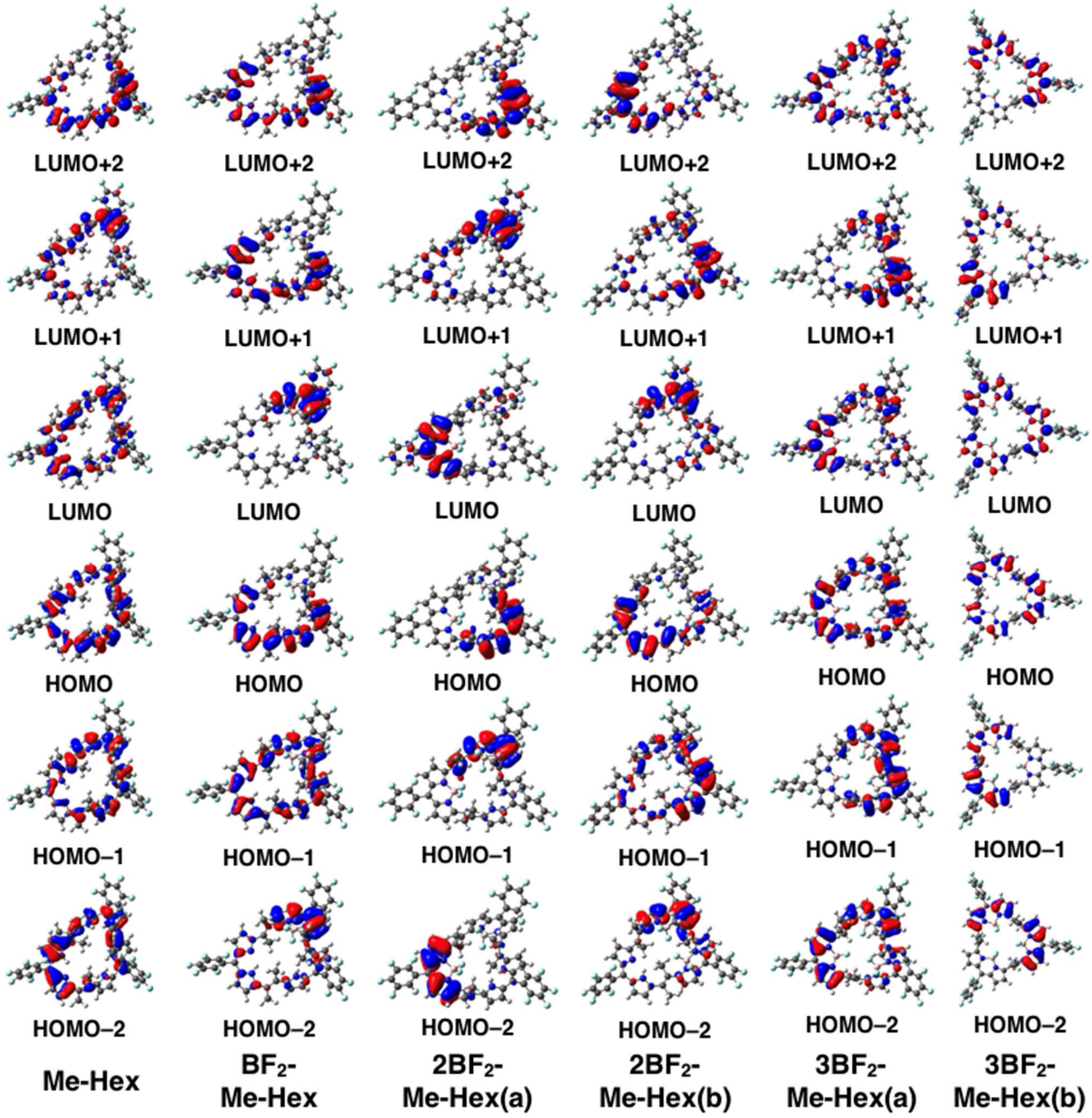

Figure S13. Cohn-Sham molecular orbitals of **Me-Hex** and boron complexes calculated at the B3LYP/6-31G* level of theory.

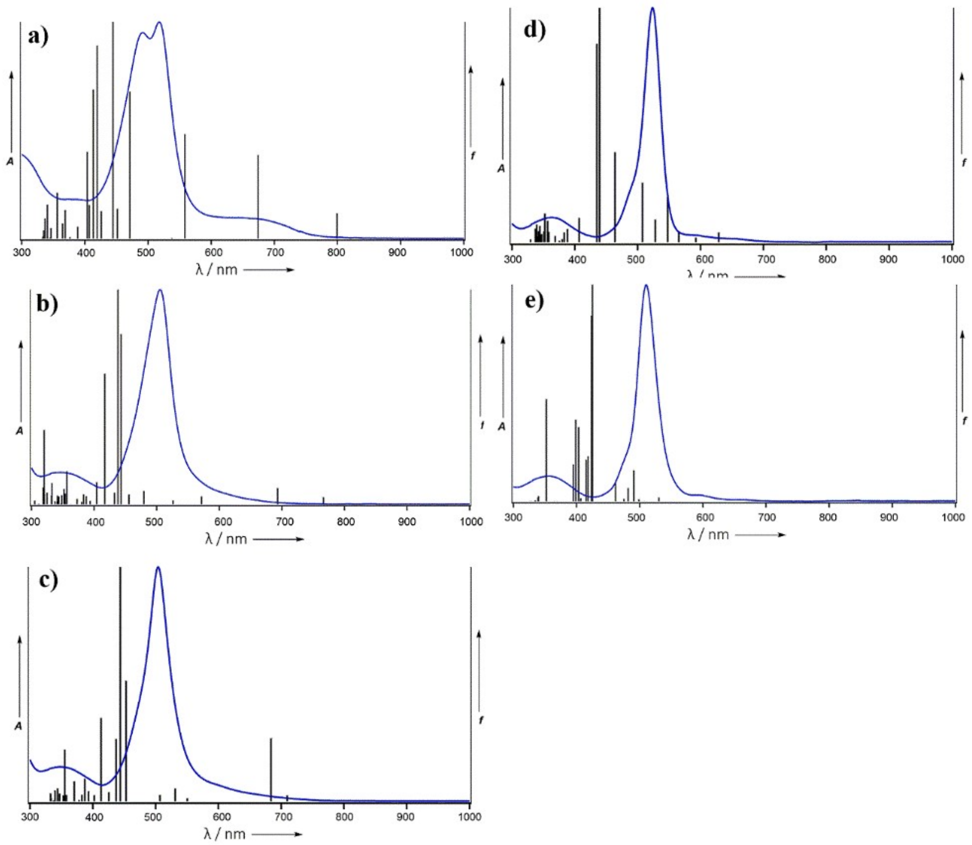

Figure S14. Simulated absorption spectra with TD-DFT of a) **BF$_2$-Me-Hex**, b) **2BF$_2$-Me-Hex(a)**, c) **2BF$_2$-Me-Hex(b)**, d) **3BF$_2$-Me-Hex(a)**, and e) **3BF$_2$-Me-Hex(b)**.

Table 1. Selected TD-DFT calculated energies, oscillator strength (*f*), and major molecular orbital contribution of **BF_2-Me-Hex**, **2BF_2-Me-Hex(a)**, **2BF_2-Me-Hex(b)**, **3BF_2-Me-Hex(a)**, and **3BF_2-Me-Hex(b)**..

Complexes	States	Wavelength (nm / eV)	*f*	Major Contributions
**BF_2-Me-Hex**	1	799.28 / 1.5512	0.0491	HOMO > LUMO
	2	537.79 / 2.3054	0.0020	HOMO > LUMO+2
**2BF_2-Me-Hex(a)**	1	766.46 / 1.6176	0.0191	HOMO > LUMO
	2	693.28 / 1.7884	0.0438	HOMO > LUMO+1
**2BF_2-Me-Hex(b)**	1	709.65 / 1.7471	0.0164	HOMO > LUMO
	2	550.70 / 2.2514	0.0088	HOMO-1 > LUMO
**3BF_2-Me-Hex(a)**	1	629.04 / 1.9710	0.0248	HOMO > LUMO
	2	592.67 / 2.0919	0.0122	HOMO-1 > LUMO
**3BF_2-Me-Hex(b)**	1	530.44 / 2.3374	0.0077	HOMO > LUMO
	2	506.91 / 2.4459	0.0022	HOMO > LUMO+1

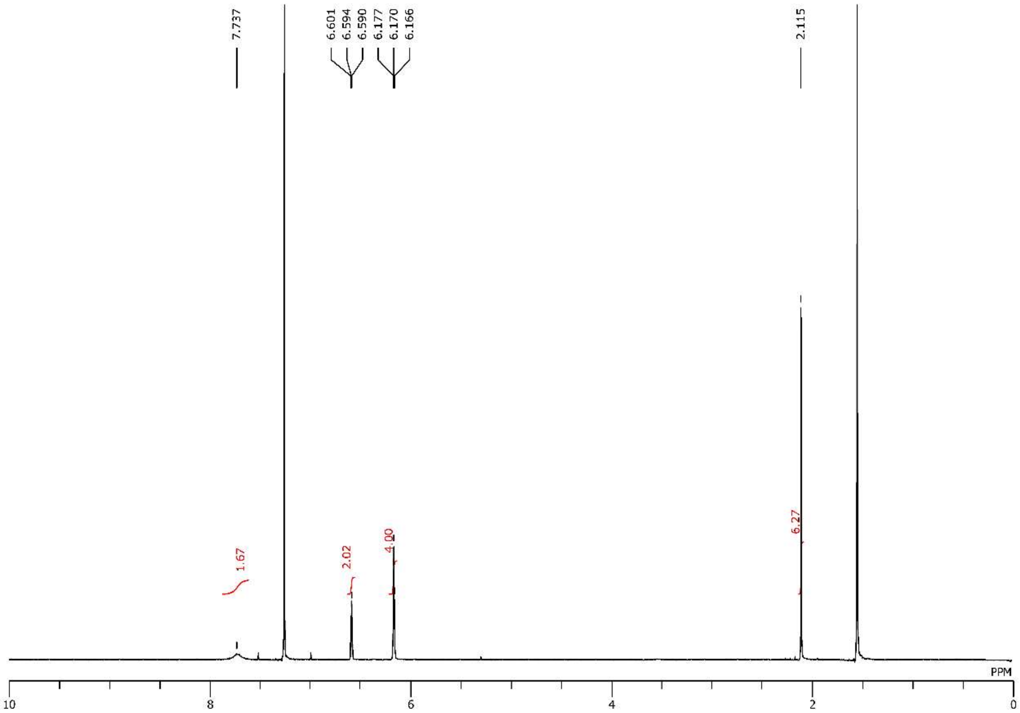

Figure S15. ^{1}H NMR spectrum of ***Z*-1** in $CDCl_3$.

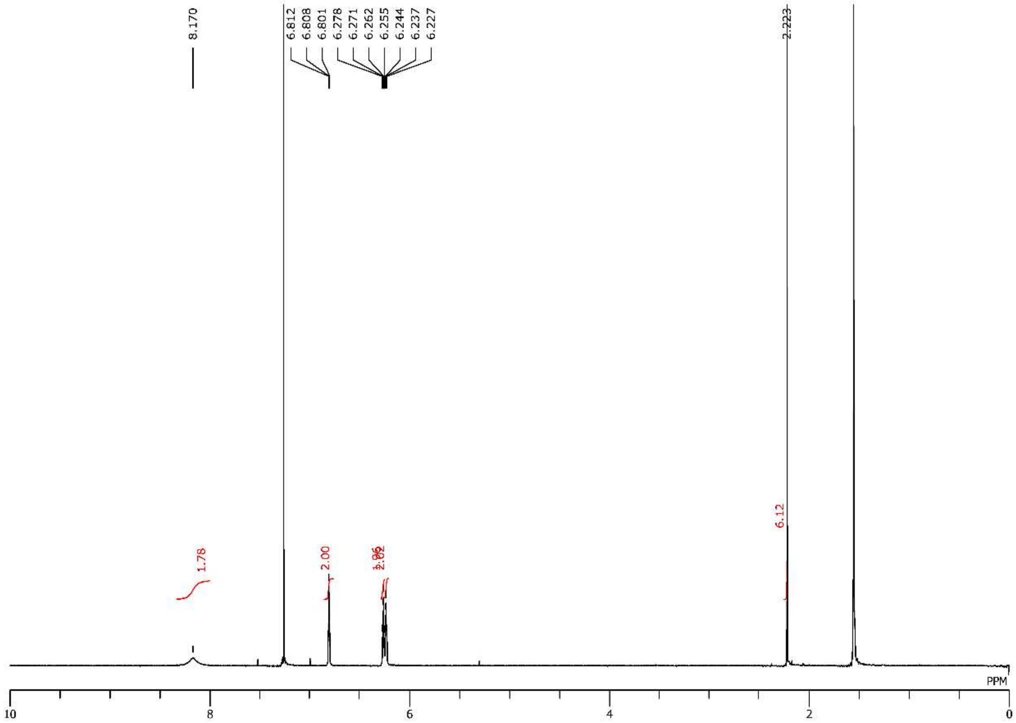

Figure S16. ^{1}H NMR spectrum of ***E*-1** in $CDCl_3$.

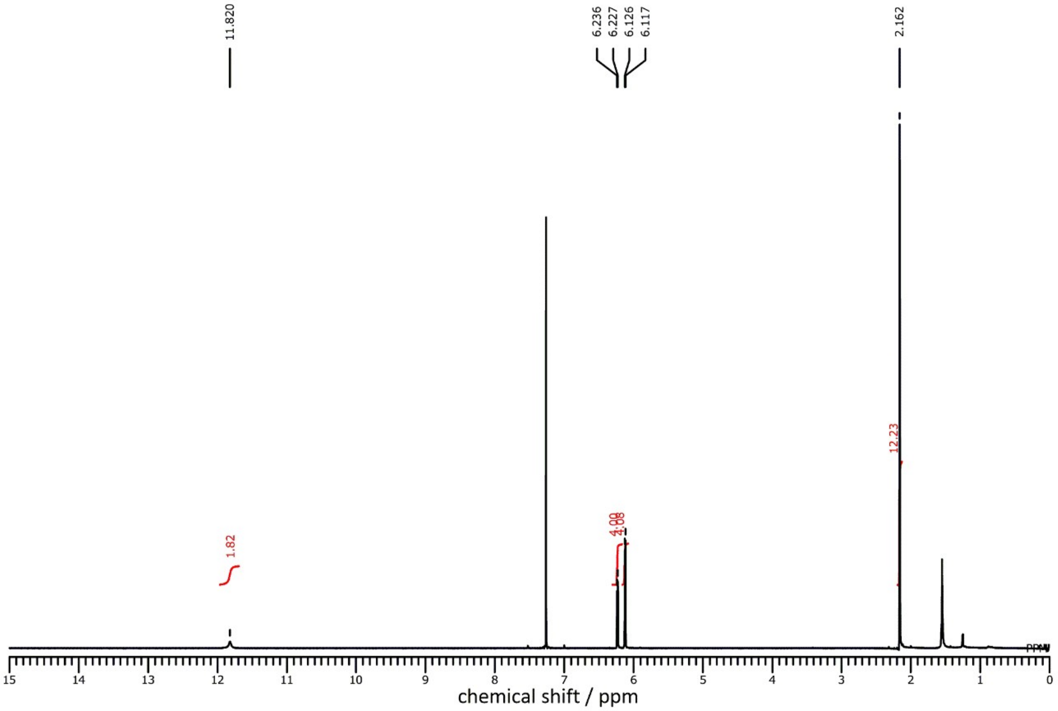

Figure S17. ^{1}H NMR spectrum of **Me-Por** in $CDCl_3$.

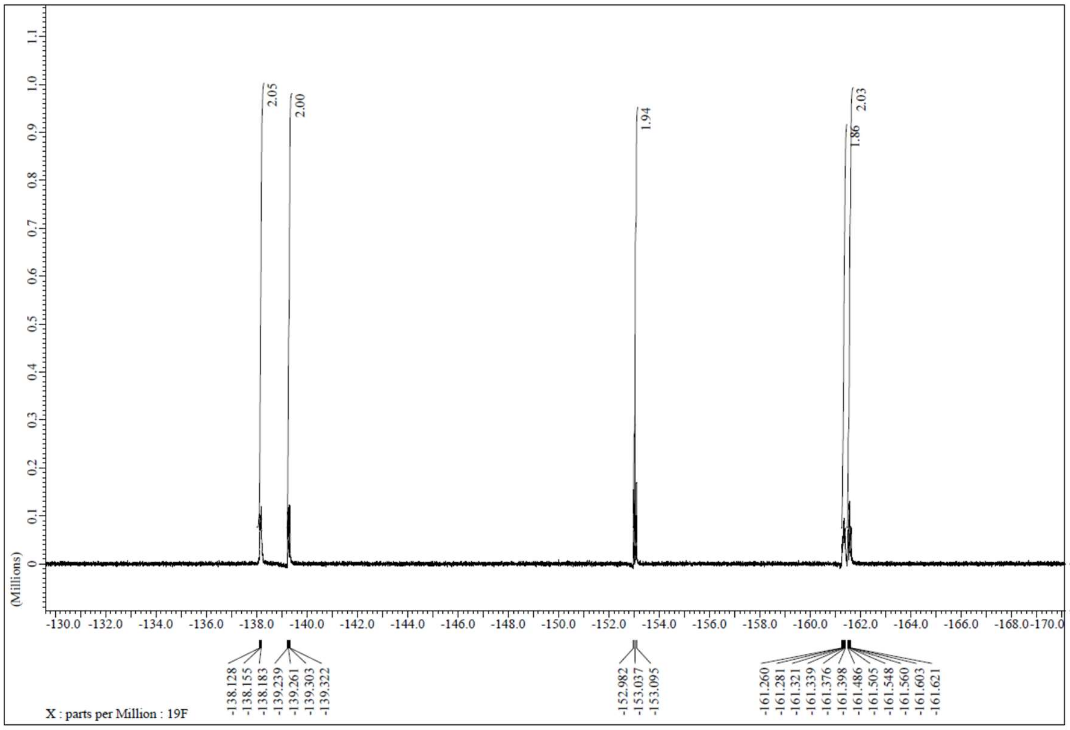

Figure 18. ^{19}F NMR spectrum of **Me-Por** in $CDCl_3$.

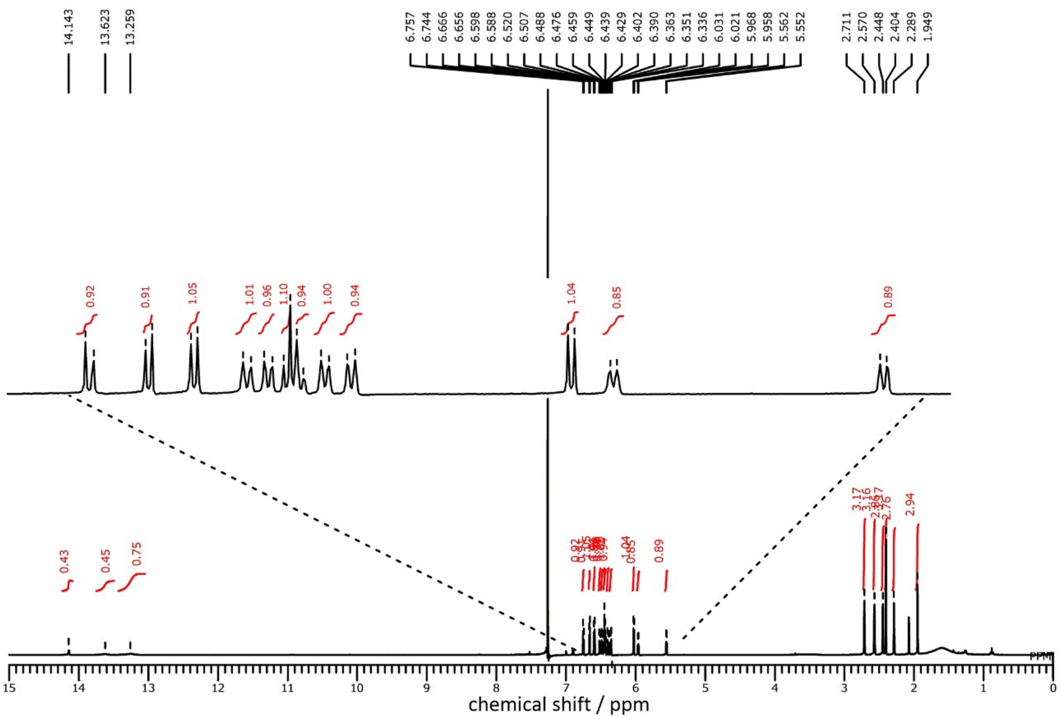

Figure S19. ^{1}H NMR spectrum of **Me-Hex** in $CDCl_3$.

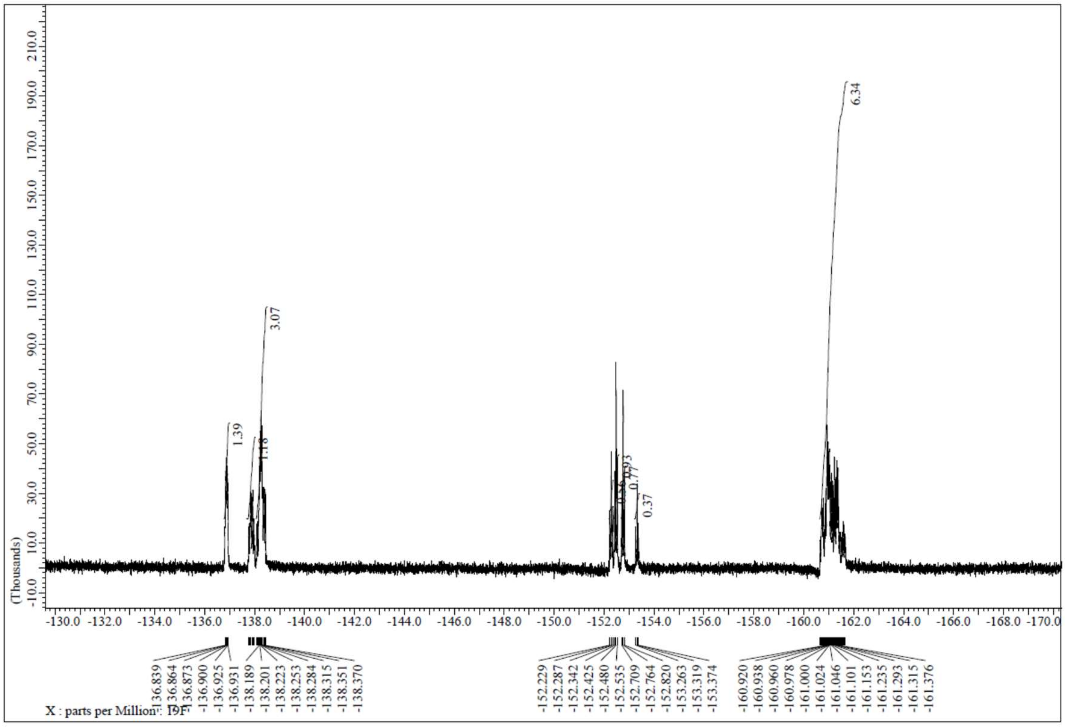

Figure S20. ^{19}F NMR spectrum of **Me-Hex** in $CDCl_3$.

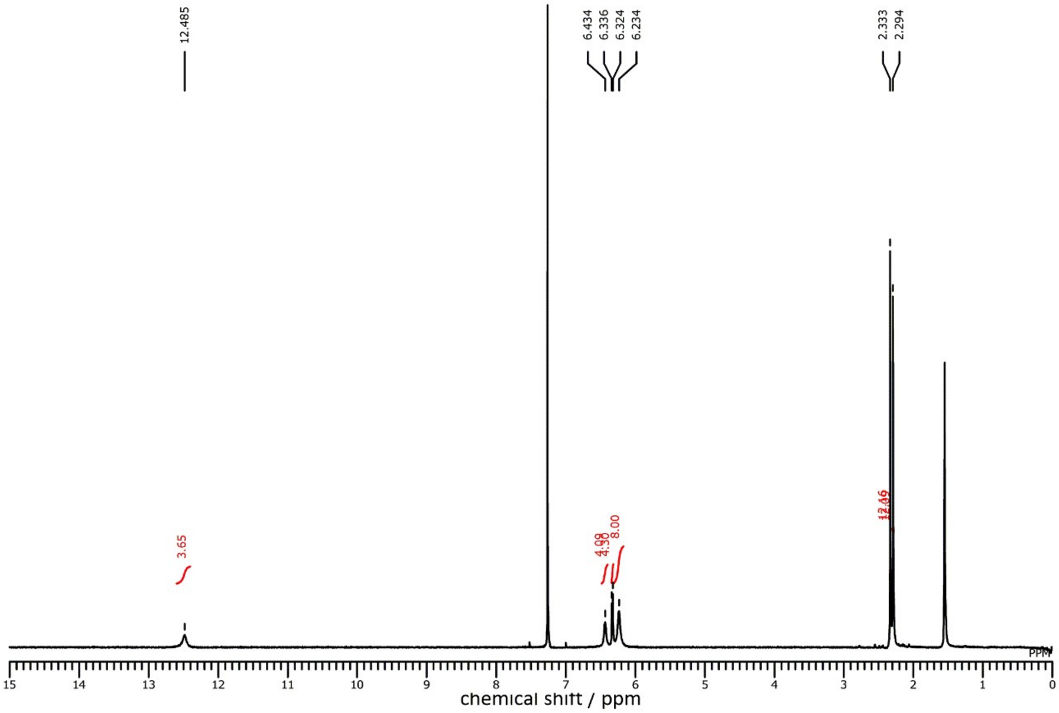

Figure S21. ^{1}H NMR spectrum of **Me-Oct** in $CDCl_3$.

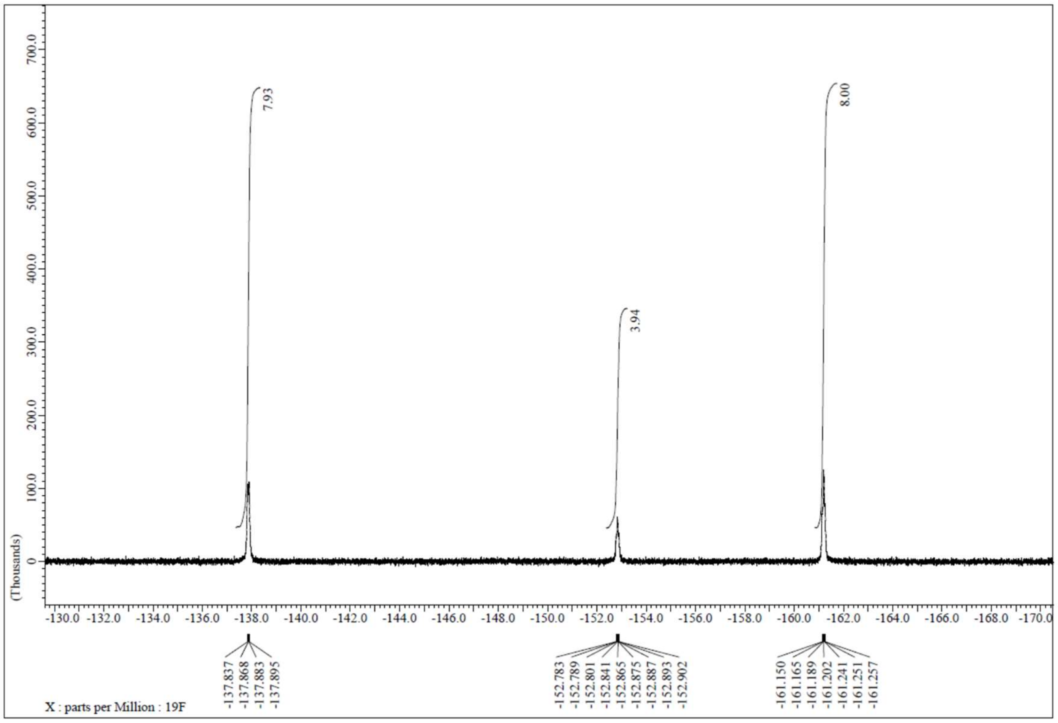

Figure S22. ^{19}F NMR spectrum of **Me-Oct** in $CDCl_3$.

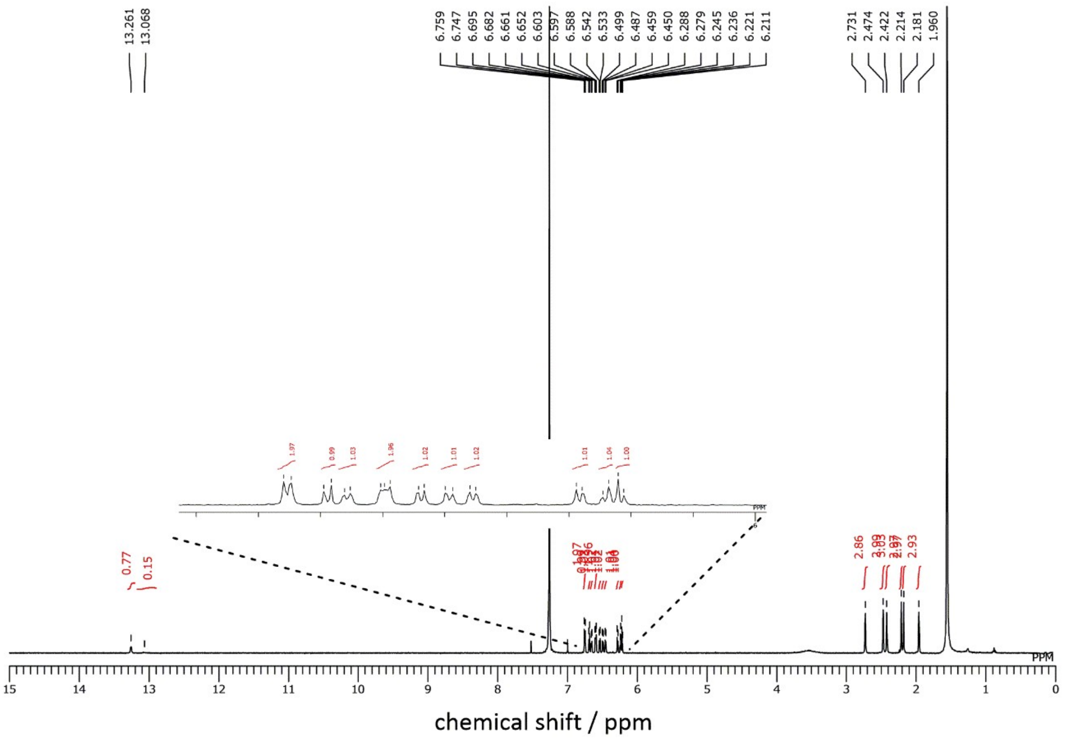

Figure S23. ^{1}H NMR spectrum of **BF2-Me-Hex** in $CDCl_3$.

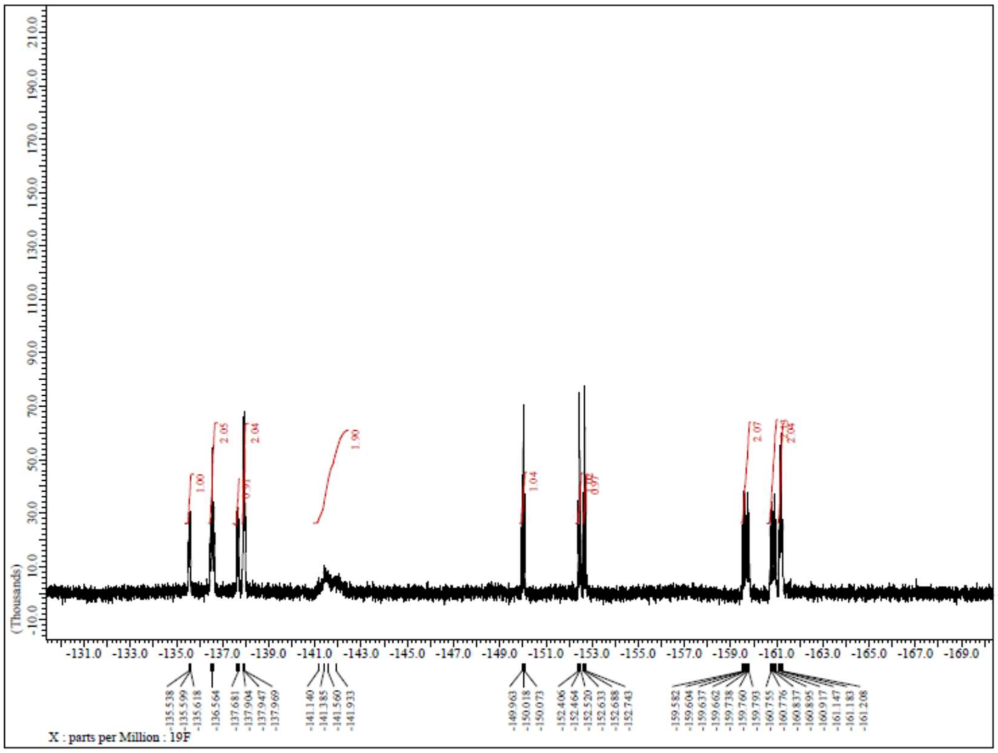

Figure S24. ^{19}F NMR spectrum of **BF2-Me-Hex** in $CDCl_3$.

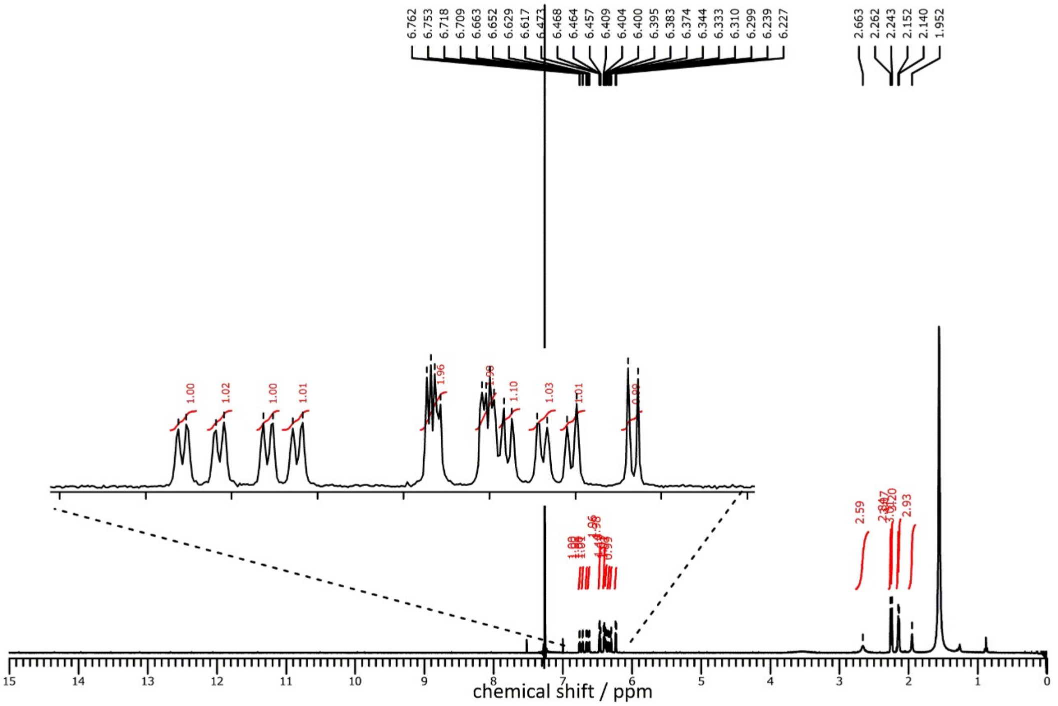

Figure S25. ^{1}H NMR spectrum of **$2BF_2$-Me-Hex(a)** in $CDCl_3$.

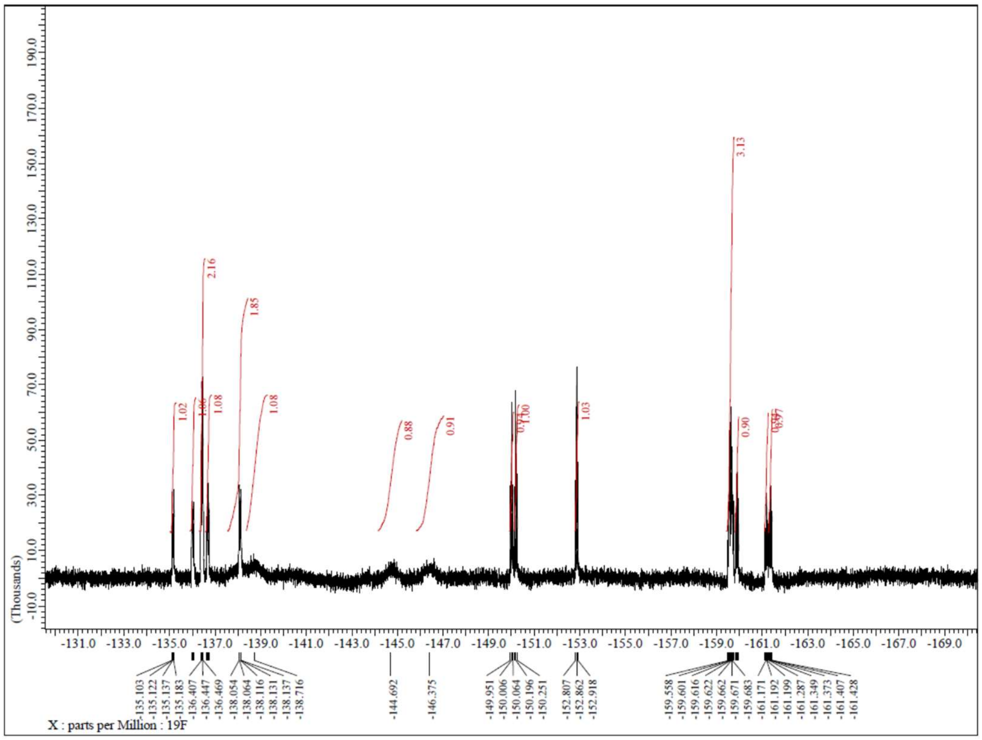

Figure S26. ^{19}F NMR spectrum of **$2BF_2$-Me-Hex(a)** in $CDCl_3$.

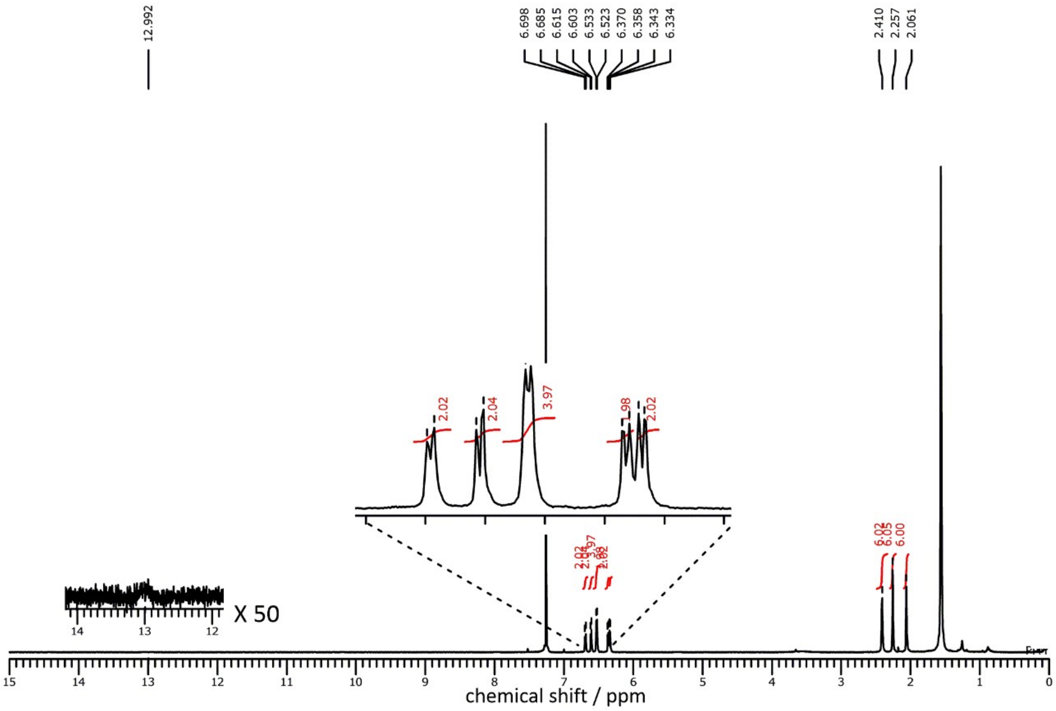

Figure S27. ^{1}H NMR spectrum of **2BF₂-Me-Hex(b)** in $CDCl_3$ at 213K.

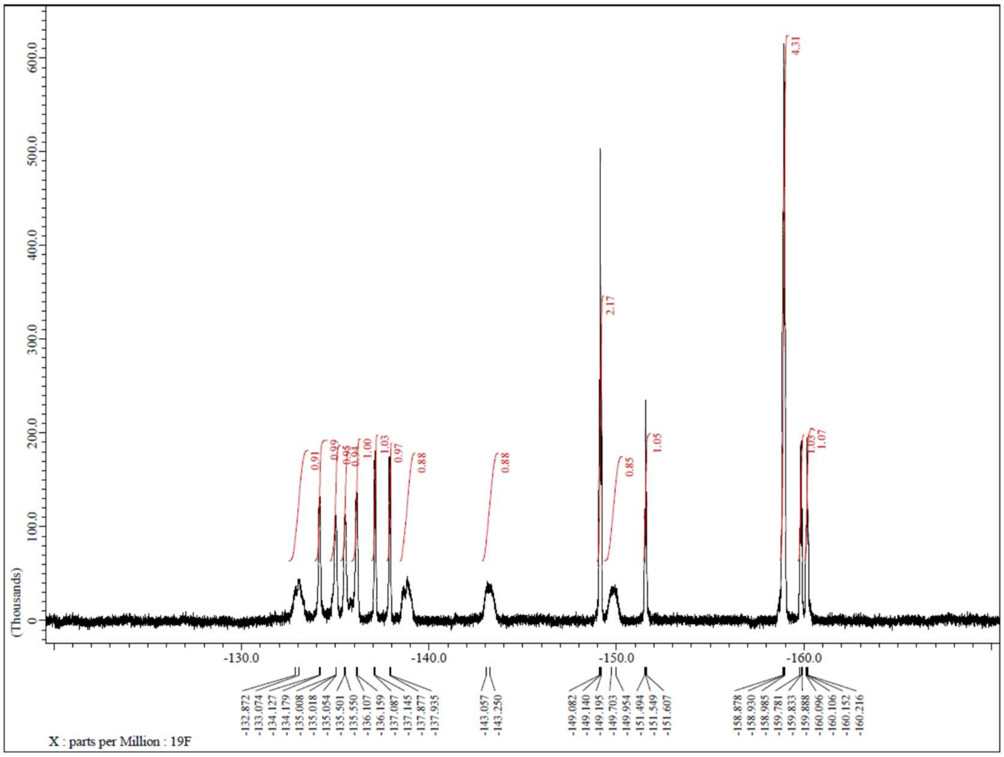

Figure S28. ^{19}F NMR spectrum of **2BF₂-Me-Hex(b)** in $CDCl_3$ at 213K.

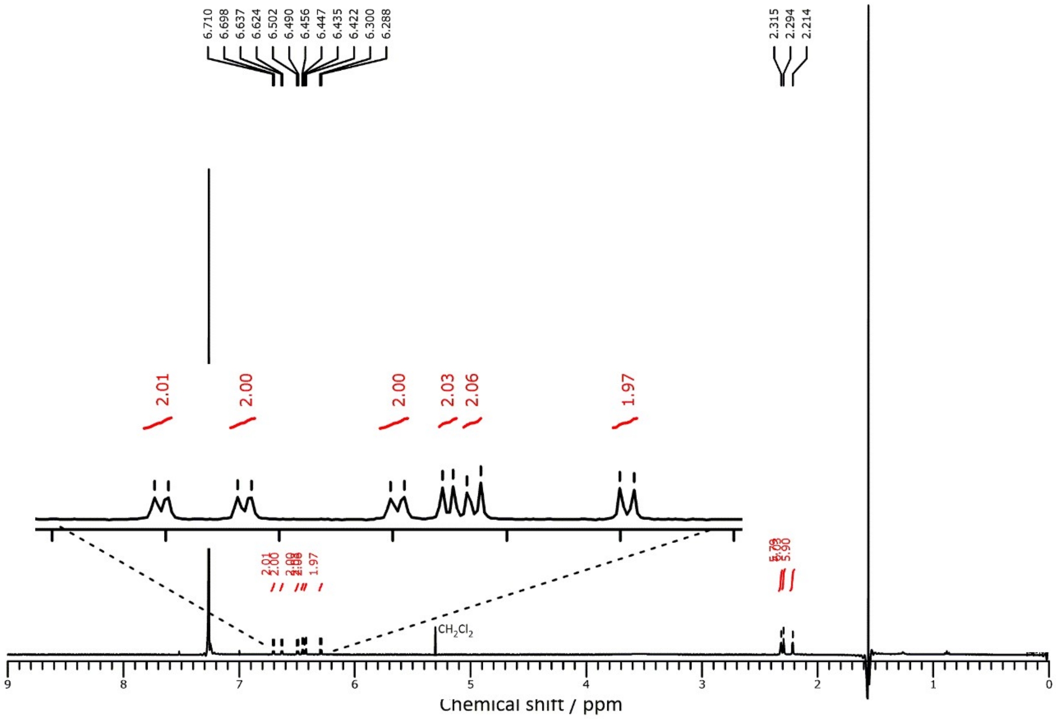

Figure S29. ^{1}H NMR spectrum of **3BF₂-Me-Hex(a)** in CDCl₃.

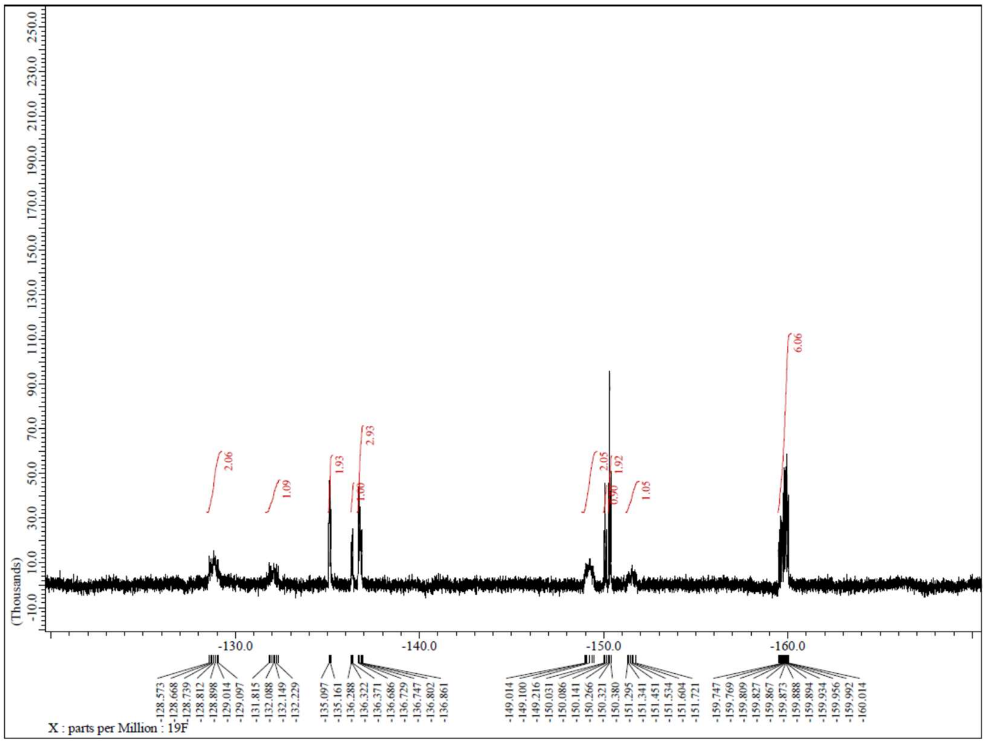

Figure S30. ^{19}F NMR spectrum of **3BF₂-Me-Hex(a)** in CDCl₃.

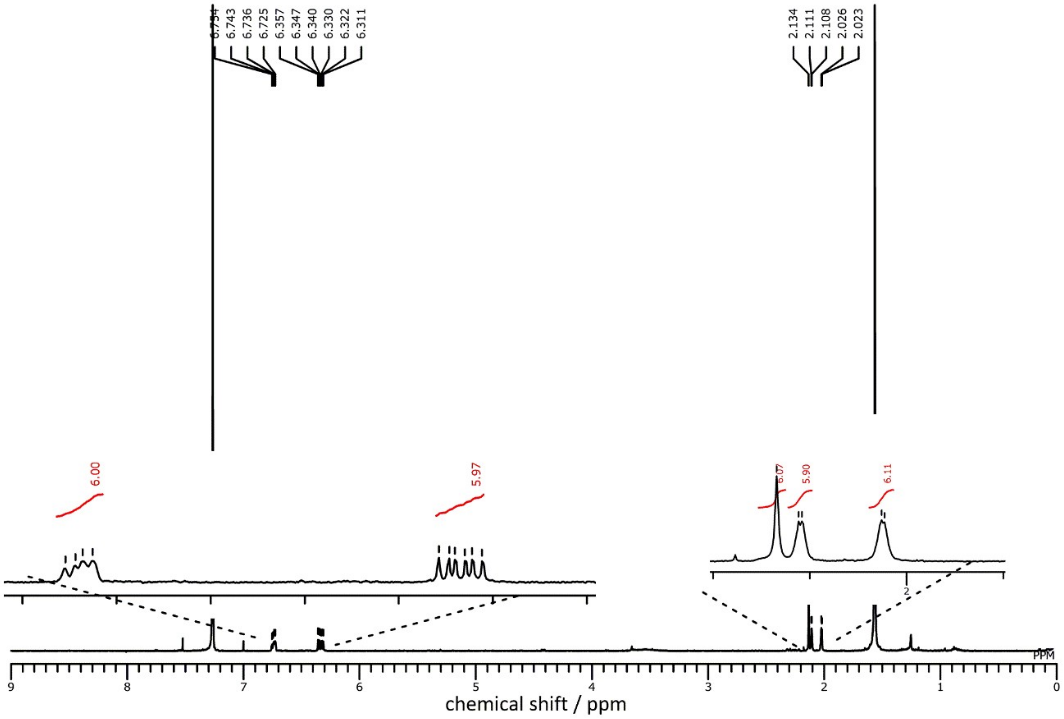

Figure S31. ^{1}H NMR spectrum of **3BF$_2$-Me-Hex(b)** in $CDCl_3$.

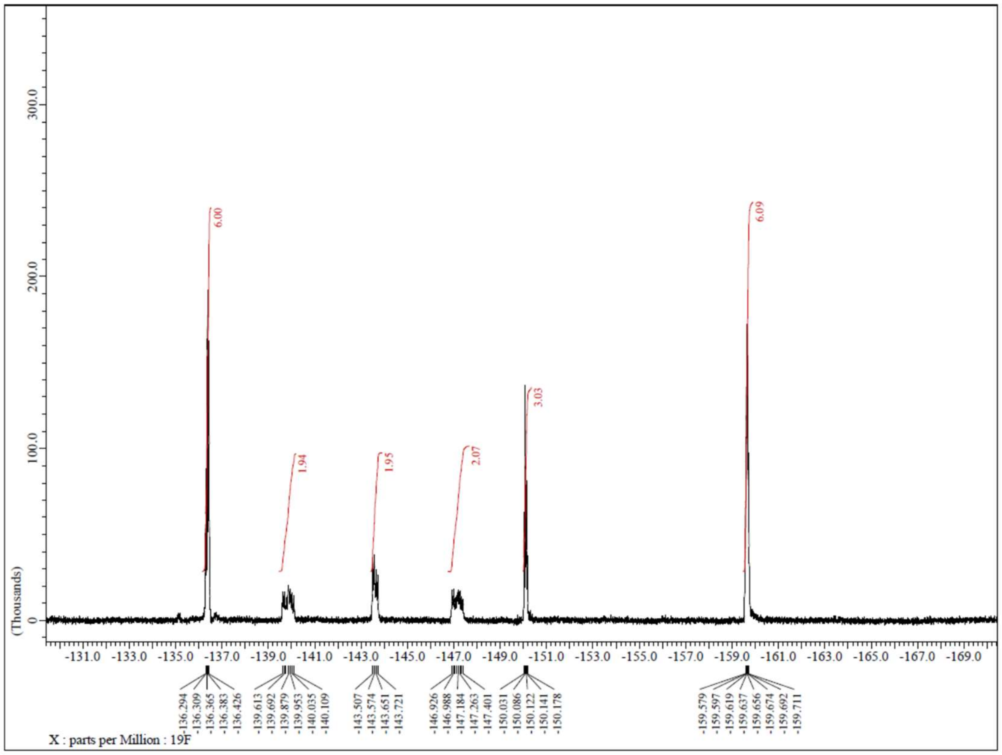

Figure S32. ^{19}F NMR spectrum of **3BF$_2$-Me-Hex(b)** in $CDCl_3$.

X-ray crystal data

Table S2. Crystal data and structure refinement of **Me-Por**.

Crystallization solvents	$CHCl_3$/Hexane	
Empirical formula	$C_{38}H_{22}F_{10}N_4 \cdot 0.5(CHCl_3)$	
Formula weight	784.28	
Temperature	103 K	
Wavelength	0.71075 Å	
Crystal system	Monoclinic	
Space group	$P2_1/c$ (#14)	
Unit cell dimensions	a = 17.4510(4) Å	
	b = 19.8253(4) Å	β = 91.195(6)°
	c = 10.1920(2) Å	
Volume	3525.37(13) Å^3	
Z	4	
Density (calculated)	1.478 g cm^{-3}	
Absorption coefficient	0.235 mm^{-1}	
F(000)	1588	
Crystal size	0.130 x 0.020 x 0.20 mm^3	
Theta range for data collection	3.080 to 25.350°	
Index ranges	$-21 \leq h \leq 21$, $-23 \leq k \leq 23$, $-12 \leq l \leq 12$	
Reflections collected	48998	
Independent reflections	6442 [R(int) = 0.0692]	
Completeness to theta = 25.242°	99.8%	
Absorption correction	Semi-empirical from equivalents	
Max. and min. transmission	0.998 and 0.801	
Refinement method	Full-matrix least-squares on F^2	
Data / restraints / parameters	6442 / 0 / 473	
Goodness-of-fit on F^2	1.017	
Final R indices [$I > 2\sigma(I)$]	R_1 = 0.0444, wR_2 = 0.0866	
R indices (all data)	R_1 = 0.0677, wR_2 = 0.0937	
Largest diff. peak and hole	0.232 and –0.214 e.Å^{-3}	

Although there are electron densities at special positions due to 0.5 molecules of chloroform, they are severely disordered and cannot be modeled properly. Therefore, we performed SQUEEZE program (solvent accessible volume: 432 Å^3, electrons found: 119 e$^-$).

Table 3. Crystal data and structure refinement of **Me-Hex.**

Crystallization solvents	CH_2Cl_2/Heptane	
Empirical formula	$C_{57}H_{33}F_{15}N_6 \cdot CH_2Cl_2$	
Formula weight	1171.82	
Temperature	103 K	
Wavelength	0.71075 Å	
Crystal system	Monoclinic	
Space group	$P2_1/c$ (#14)	
Unit cell dimensions	a = 10.0477(2) Å	
	b = 18.2174(5) Å	β = 99.059(7)°
	c = 28.7465(7) Å	
Volume	5196.2(2) $Å^3$	
Z	4	
Density (calculated)	1.498 g cm^{-3}	
Absorption coefficient	0.226 mm^{-1}	
F(000)	2376	
Crystal size	0.050 x 0.040 x 0.030 mm^3	
Theta range for data collection	3.036 to 24.404°	
Index ranges	$-11 \le h \le 11$, $-21 \le k \le 21$, $-33 \le l \le 33$	
Reflections collected	64768	
Independent reflections	8541 [R(int) = 0.0929]	
Completeness to theta = 24.404°	99.8%	
Absorption correction	Semi-empirical from equivalents	
Max. and min. transmission	0.996 and 0.665	
Refinement method	Full-matrix least-squares on F^2	
Data / restraints / parameters	8541 / 0 / 709	
Goodness-of-fit on F^2	1.030	
Final R indices [$I > 2\sigma(I)$]	R_1 = 0.0571, wR_2 = 0.1263	
R indices (all data)	R_1 = 0.0865, wR_2 = 0.1386	
Largest diff. peak and hole	0.321 and –0.278 e.$Å^{-3}$	

Although there are electron densities around molecules due to a molecule of dichloromethane, they are severely disordered and cannot be modeled properly. Therefore, we performed SQUEEZE program (solvent accessible volume: 720 $Å^3$, electrons found: 148 e^-).

Table S4. Crystal data and structure refinement of **Me-Oct.**

Crystallization solvents	Toluene
Empirical formula	$C_{76}H_{44}F_{20}N_8 \cdot 2(C_7H_8)$
Formula weight	1633.49
Temperature	103 K
Wavelength	0.71075 Å
Crystal system	Monoclinic
Space group	$P2_1/c$ (#14)
Unit cell dimensions	a = 9.2800(7) Å
	b = 34.913(3) Å β = 97.438(7)°
	c = 23.6880(18) Å
Volume	7610.1(10) $Å^3$
Z	4
Density (calculated)	1.426 g cm^{-3}
Absorption coefficient	0.1186 mm^{-1}
F(000)	3344.00
Crystal size	0.260 x 0.030 x 0.020 mm^3
Theta range for data collection	3.035 to 24.403°
Index ranges	$-10 \le h \le 10$, $-40 \le k \le 40$, $-27 \le l \le 27$
Reflections collected	94248
Independent reflections	12440 [R(int) = 0.3660]
Completeness to theta = 24.403°	99.4%
Absorption correction	Semi-empirical from equivalents
Max. and min. transmission	0.998 and 0.356
Refinement method	Full-matrix least-squares on F^2
Data / restraints / parameters	12440 / 0 / 1073
Goodness-of-fit on F^2	1.005
Final R indices [$I > 2\sigma(I)$]	R_1 = 0.1115, wR_2 = 0.2430
R indices (all data)	R_1 = 0.2558, wR_2 = 0.3187
Largest diff. peak and hole	0.42 and –0.31 e.$Å^{-3}$

Despite many attempts, we could obtain only thin needle crystals. For the relatively large unit cell, the solvent-containing crystal does not contain any heavy atoms. Therefore, no high-angle diffractions were obtained and R-value got high.

Table S5. Crystal data and structure refinement of **BF_2-Me-Hex.**

Crystallization solvents	$CHCl_3$/Toluene/Hexane	
Empirical formula	$C_{57}H_{32}BF_{17}N_6 \cdot 2(CHCl_3)$	
Formula weight	1373.43	
Temperature	90 K	
Wavelength	0.71073 Å	
Crystal system	Triclinic	
Space group	*P*-1 (#2)	
Unit cell dimensions	a = 13.267(2) Å	α = 104.422(3)°
	b = 15.334(3) Å	β = 109.003(3)°
	c = 16.490(3) Å	γ = 99.003(3)°
Volume	2966.7(9) $Å^3$	
Z	2	
Density (calculated)	1.538 g cm^{-3}	
Absorption coefficient	0.389 mm^{-1}	
F(000)	1380	
Crystal size	0.300 x 0.100 x 0.020 mm^3	
Theta range for data collection	1.631 to 24.000°	
Index ranges	$-15 \leq h \leq 15$, $-11 \leq k \leq 17$, $-18 \leq l \leq 18$	
Reflections collected	14415	
Independent reflections	9245 [*R*(int) = 0.0395]	
Completeness to theta = 24.000°	99.2%	
Absorption correction	Semi-empirical from equivalents	
Max. and min. transmission	0.992 and 0.812	
Refinement method	Full-matrix least-squares on F^2	
Data / restraints / parameters	9245 / 96 / 846	
Goodness-of-fit on F^2	1.029	
Final *R* indices [I>2$\sigma(I)$]	R_1 = 0.0639, wR_2 = 0.1359	
R indices (all data)	R_1 = 0.1229, wR_2 = 0.1524	
Largest diff. peak and hole	0.520 and –0.290 e.$Å^{-3}$	

Although there are electron densities around molecules due to two molecules of chloroform, they are severely disordered and cannot be modeled properly. Therefore, we performed SQUEEZE program (solvent accessible volume: 705 $Å^3$, electrons found: 227 e^-).

One vinylene unit was analyzed as a disordered part. SIMU restraints were performed on the minor part of disordered vinylene unit.

Table S6. Crystal data and structure refinement of **$2BF_2$-Me-Hex(a).**

Crystallization solvents	$CHCl_3$/Hexane	
Empirical formula	$C_{57}H_{31}B_2F_{19}N_6$	
Formula weight	1182.50	
Temperature	103 K	
Wavelength	0.71075 Å	
Crystal system	monoclinic	
Space group	$P2_1/c$ (#14)	
Unit cell dimensions	a = 14.1364(9) Å	
	b = 30.887(2) Å	β = 103.727(7)°
	c = 11.9832(8) Å	
Volume	5082.7(6) $Å^3$	
Z	4	
Density (calculated)	1.545 g cm^{-3}	
Absorption coefficient	0.1403 mm^{-1}	
F(000)	2384.00	
Crystal size	0.200 x 0.100 x 0.010 mm^3	
Theta range for data collection	3.027 to 25.351°	
Index ranges	$-17 \le h \le 16$, $-37 \le k \le 37$, $-14 \le l \le 14$	
Reflections collected	68779	
Independent reflections	9295 [R(int) = 0.1627]	
Completeness to theta = 25.242°	99.8%	
Absorption correction	Semi-empirical from equivalents	
Max. and min. transmission	0.999 and 0.212	
Refinement method	Full-matrix least-squares on F^2	
Data / restraints / parameters	9295 / 0 / 763	
Goodness-of-fit on F^2	1.043	
Final R indices [$I > 2\sigma(I)$]	R_1 = 0.0976, wR_2 = 0.2241	
R indices (all data)	R_1 = 0.1767, wR_2 = 0.2668	
Largest diff. peak and hole	0.282 and –0.257 e.$Å^{-3}$	

Table S7. Crystal data and structure refinement of **2BF$_2$-Me-Hex(b).**

Crystallization solvents	$CHCl_3$/Hexane	
Empirical formula	$C_{57}H_{31}B_2F_{19}N_6 \cdot 1.5(C_6H_{14})$	
Formula weight	1311.75	
Temperature	90 K	
Wavelength	0.71073 Å	
Crystal system	Triclinic	
Space group	*P*-1 (#2)	
Unit cell dimensions	a = 11.7169(7) Å	α = 95.7087(11)°
	b = 14.6385(9) Å	β = 97.4311(12)°
	c = 18.5296(11) Å	γ = 105.6457(11)°
Volume	3004.5(3) Å^3	
Z	2	
Density (calculated)	1.450 g cm^{-3}	
Absorption coefficient	0.127 mm^{-1}	
F(000)	1342	
Crystal size	0.300 x 0.300 x 0.300 mm^3	
Theta range for data collection	1.983 to 27.939°	
Index ranges	$-15 \le h \le 12$, $-19 \le k \le 19$, $-16 \le l \le 24$	
Reflections collected	20417	
Independent reflections	14252 [R(int) = 0.0186]	
Completeness to theta = 25.242°	99.3%	
Absorption correction	Semi-empirical from equivalents	
Max. and min. transmission	0.963 and 0.907	
Refinement method	Full-matrix least-squares on F^2	
Data / restraints / parameters	14252 / 104 / 945	
Goodness-of-fit on F^2	1.014	
Final R indices [I>2σ(I)]	R_1 = 0.0752, wR_2 = 0.2397	
R indices (all data)	R_1 = 0.1107, wR_2 = 0.2854	
Largest diff. peak and hole	1.088 and –0.715 e.Å^{-3}	

One vinylene-dipyrrin unit was analyzed as a disordered part. Appropriate SIMU, ISOR, and SADI restraints were performed on the disordered unit.

Table S8. Crystal data and structure refinement of **3BF$_2$-Me-Hex(a).**

Crystallization solvents	$CHCl_3$/Hexane
Empirical formula	$C_{57}H_{29}B_3F_{21}N_6 \cdot 3(CHCl_3)$
Formula weight	1587.39
Temperature	103 K
Wavelength	0.71075 Å
Crystal system	Trigonal
Space group	*P*3*c*1 (#158)
Unit cell dimensions	*a* = 26.8942(12) Å
	c = 15.0541(3) Å
Volume	9429.8(6) Å^3
Z	6
Density (calculated)	1.677 g cm^{-3}
Absorption coefficient	0.510 mm^{-1}
F(000)	4746.00
Crystal size	0.120 x 0.050 x 0.050 mm^3
Theta range for data collection	2.624 to 25.343°
Index ranges	$-32 \le h \le 32$, $-32 \le k \le 32$, $-18 \le l \le 16$
Reflections collected	125837
Independent reflections	11291 [*R*(int) = 0.1164]
Completeness to theta = 25.242°	99.9%
Absorption correction	Semi-empirical from equivalents
Max. and min. transmission	0.975 and 0.497
Refinement method	Full-matrix least-squares on F^2
Data / restraints / parameters	11291 / 1 / 911
Goodness-of-fit on F^2	1.285
Final *R* indices [$I > 2\sigma(I)$]	R_1 = 0.1228, wR_2 = 0.3120
R indices (all data)	R_1 = 0.1434, wR_2 = 0.3267
Absolute structure parameter	0.19(3)
Largest diff. peak and hole	1.712 and –0.701 e.Å^{-3}

One of three chloroforms was analyzed as a disordered molecule.

Table S9. Crystal data and structure refinement of **3BF$_2$-Me-Hex(b).**

Crystallization solvents	$CHCl_3$/Hexane
Empirical formula	$C_{57}H_{30}B_3F_{21}N_6 \cdot 3(CHCl_3)$
Formula weight	1588.40
Temperature	90 K
Wavelength	0.71073 Å
Crystal system	Hexagonal
Space group	$P6_3/m$ (#176)
Unit cell dimensions	a = 20.714(3) Å
	c = 9.1709(12) Å
Volume	3407.7(10) Å^3
Z	2
Density (calculated)	1.548 g cm^{-3}
Absorption coefficient	0.471 mm^{-1}
F(000)	1584
Crystal size	0.200 x 0.100 x 0.050 mm^3
Theta range for data collection	1.966 to 24.976°
Index ranges	$-24 \le h \le 21$, $-24 \le k \le 20$, $-10 \le l \le 10$
Reflections collected	17971
Independent reflections	2124 [R(int) = 0.0673]
Completeness to theta = 24.976°	99.8%
Absorption correction	Semi-empirical from equivalents
Max. and min. transmission	0.977 and 0.846
Refinement method	Full-matrix least-squares on F^2
Data / restraints / parameters	2124 / 10 / 247
Goodness-of-fit on F^2	1.054
Final R indices [I>2σ(I)]	R_1 = 0.1073, wR_2 = 0.2947
R indices (all data)	R_1 = 0.1569, wR_2 = 0.3702
Largest diff. peak and hole	0.910 and –0.497 e.Å^{-3}

The asymmetric unit was analyzed by placing one-sixth of the whole molecular structure in a special position.
